# A Systematic Review and Meta-Analysis of Human Milk Feeding and Short-Term Growth in Preterm and Very Low Birth Weight Infants

**DOI:** 10.3390/nu13062089

**Published:** 2021-06-18

**Authors:** Machiko Suganuma, Alice R. Rumbold, Jacqueline Miller, Yan Fong Chong, Carmel T. Collins

**Affiliations:** 1 SAHMRI Women and Kids, South Australian Health and Medical Research Institute, Adelaide, SA 5006, Australia; sakai197723@yahoo.co.jp (M.S.); alice.rumbold@sahmri.com (A.R.R.); jacqueline.miller@sahmri.com (J.M.); 2 Adelaide Medical School, The University of Adelaide, Adelaide, SA 5006, Australia; 3 College of Nursing and Health Sciences, Flinders University, Adelaide, SA 5001, Australia; chong.yanfong@gmail.com

**Keywords:** preterm infant, human milk, growth

## Abstract

Human milk (HM) is the gold standard for feeding infants but has been associated with slower growth in preterm infants compared with preterm formula. This systematic review and meta-analysis summarises the post-1990 literature to examine the effect of HM feeding on growth during the neonatal admission of preterm infants with birth weight ≤1500 g and/or born ≤28 weeks’ gestation. Medline, PubMed, CINAHL, and Scopus were searched, and comparisons were grouped as exclusive human milk (EHM) vs. exclusive preterm formula (EPTF), any HM vs. EPTF, and higher vs. lower doses of HM. We selected studies that used fortified HM and compared that with a PTF; studies comparing unfortified HM and term formula were excluded. Experimental and observational studies were pooled separately. The GRADE system was used to evaluate risk of bias and certainty of evidence. Forty-four studies were included with 37 (*n* = 9963 infants) included in the meta-analyses. In general, due to poor quality studies, evidence of the effect of any HM feeds or higher versus lower doses of HM was inconclusive. There was a possible effect that lower doses of HM compared with higher doses of HM improved weight gain during the hospital admission, and separately, a possible effect of increased head circumference growth in infants fed EPTF vs. any HM. The clinical significance of this is unclear. There was insufficient evidence to determine the effects of an exclusive HM diet on any outcomes.

## 1. Introduction

Mother’s own milk (MOM) is the feed of choice for preterm infants [1] because of clear advantages for immunological, gastrointestinal, and neurodevelopmental health and function [2,3]. Where there is insufficient MOM, current recommendations are to use appropriately screened and pasteurised donor human milk (DHM) if available, and then preterm formula (PTF) [2,4]. However, human milk (HM) alone is insufficient to support the growth requirements of preterm and very low birth weight (<1500 g, VLBW) infants, with many early studies reporting poorer growth in infants fed unfortified HM than infants fed PTF [5]. Hence, nutrient fortification of HM is now standard clinical practice for VLBW babies in many settings [6]. Even with routine fortification of HM, there is inconsistent evidence about the impact of HM feeds on infant growth. For example, some studies have reported slower weight, length, and head circumference (HC) gain, whereas others suggest HM (MOM/DHM) adequately supports early postnatal growth compared with formula feeding [7,8,9]. A recent Cochrane systematic review failed to identify any randomised trials that specifically examined HM feeds versus preterm formula in preterm or low birth weight infants [10]. In another Cochrane review of the same population comparing formula versus DHM, greater short-term growth was shown in infants fed formula, even where DHM had been fortified [11]. However, there is also evidence that HM supports better quality growth, as formula-fed preterm infants have increased fat mass at term corrected age when compared to HM-fed infants [12].

Given the conflicting evidence, a greater understanding of how HM feeding, whether with MOM or DHM, affects growth in preterm and VLBW infants is necessary. This is particularly important as accumulated growth deficits in the neonatal period are associated with unfavourable neurodevelopmental outcomes in later life [13]. This review builds on our previous review of human milk feeding and morbidity in VLBW infants [3] and aims to provide a direct comparison of growth between infants fed exclusive PTF (EPTF) and exclusive HM (EHM) and to explore the associations with various doses of HM intake and growth. We summarise the evidence on growth, i.e., weight, length, and HC gain, and body composition (proportion of fat mass and fat-free mass). The present review provides a comprehensive overview inclusive of both randomised controlled trials (RCTs) and, given the limited number of trials, observational studies, and varying doses of HM feeding, reflective of current practice for VLBW infants.

## 2. Materials and Methods

### 2.1. Registration and Reporting

This review is registered with PROSPERO International prospective register of systematic reviews (PROSPERO 2018 CRD42018104389) and the protocol is available from http://www.crd.york.ac.uk/PROSPERO/display_record.php?ID=CRD42018104389 (accessed on 16 June 2021). The Preferred Reporting Items for Systematic Reviews and Meta-Analyses (PRSMA) [14] statement has been followed.

### 2.2. Eligibility Criteria

#### 2.2.1. Types of Studies

RCTs and observational studies, published from 1990 onwards, were considered for inclusion in this review. All component studies of relevant systematic reviews were also considered. Secondary analyses of RCTs were considered as cohort studies. Comparisons between groups in RCTs that included a non-randomised arm, usually a reference feeding group, were considered as a cohort design (e.g., Costa-Orvay, 2011 [15]).

#### 2.2.2. Types of Participants

Infants born at ≤28 weeks’ gestation and/or study populations with a reported mean birth weight ≤1500 g were considered for inclusion. Post-discharge studies were excluded. As quantifying the exposure of HM was necessary for group studies, we excluded studies where this could not be done reliably, e.g., where feeding intake data were recalled retrospectively or measured at only one time point and extrapolated for the neonatal admission.

#### 2.2.3. Types of Intervention

Studies comparing the effects of HM were grouped according to the following exposure categories:

EHM compared with EPTF. This provides a direct evaluation of the effect of the two opposite feeding methods.

Any HM (includes EHM or HM plus PTF) compared with EPTF. This explores the effect of any HM when infants also receive PTF.

Higher-dose HM (includes EHM plus high dose of HM with PTF) was compared with low-dose HM (lower dose of HM with some PTF). This comparison was included to explore the dose-related effect of HM. No a priori categories were defined for a ‘higher’ or ‘lower’ dose of HM. Therefore, studies included here may have compared EHM with mixed feeding groups, or alternatively, all infant groups may have been mixed fed, with those having a higher proportion of enteral intake as HM compared with those having a lower proportion of enteral intake as HM.

As this review is intended to reflect contemporary feeding practices, we selected studies that used fortified HM and compared that with a PTF. Studies comparing unfortified HM and term formula were excluded.

#### 2.2.4. Type of Outcome Measure

Outcome measures included:

In-hospital growth (weight, length, and HC gains), where growth rates during the neonatal admission were measured. Where findings were expressed in similar units (i.e., g/kg/d, g/d, cm/wk, z-scores), they were combined in meta-analyses.

In-hospital body composition (fat mass, lean mass, grams, and the relative percentages). Results at the study’s end were combined in meta-analyses.

In-hospital growth (i.e., during the neonatal admission) was chosen as the most appropriate period to study, as it is commonly reported and has previously been identified as a sensitive period influencing later neurodevelopmental outcomes [16]. Considerable variation in reporting infant growth has been described [17], particularly regarding weight gain, which is variously reported in g/kg/d, g/d, or z-score. There is also a lack of standardisation over the measurement period including birth to discharge or some other common start or endpoint such as from when birth weight is regained or 36 weeks post menstrual age. We report growth velocity and z-score change to show growth status changes over time [17]. Body composition measures also vary considerably in the measurement tools used, i.e., DEXA, air displacement plethysmography, and bioelectrical impedance. We have taken a broad and inclusive approach to these variations in an attempt to identify as much literature as possible. Measurement details have been documented, only data with similar units have been combined in meta-analyses, and where results for two different time points have been reported, the time point that is closest to the other studies included has been used for the meta-analyses.

### 2.3. Information Sources and Study Selection

Searches were conducted in June 2020 in the following databases: Medline (Ovid), PubMed, Scopus, The Cochrane Central Register of Controlled Trials (Wiley), and CINAHL (EBSCOhost). Results were restricted to English language published from 1990.

Search terms for each key concept (preterm infant, HM feeding, weight/length/HC gain) included database-specific broad subject terms, e.g., CINAHL Headings in CINAHL and MeSH in Cochrane Library and PubMed, and a wide range of synonyms and free-text terms were searched as text words. Necrotising enterocolitis was included as a search keyword to include studies that may report growth as a secondary outcome. Reference lists of articles were hand-searched to identify further relevant articles. Citations were exported to Covidence [18] for organisation and screening. Two authors independently screened articles against the eligibility criteria for inclusion. Disagreements were resolved by discussion between the two authors, with a third author involved if necessary.

### 2.4. Data Extraction, Risk of Bias in Individual Studies, and Data Synthesis

Two authors extracted the relevant data into tables. Data extracted were limited to comparisons typical of standard clinical care, e.g., feeding PTF with HM fortified with a multicomponent fortifier. Therefore, arms of studies where an experimental formula was used (e.g., with the addition of long-chain polyunsaturated fatty acids as in Fewtrell 2002 [19] and O’Connor 2001 [20]) were excluded, as were arms of studies using HM fortified with minerals alone [21]. In addition, the data extracted were aligned with our gestational age and birth-weight criteria. If a study included a wider gestational age and birth-weight range but reported relevant data as a subgroup that met our criteria, such as Lok 2017 [22], we extracted only those data.

The risk of bias was assessed using the Cochrane Risk of Bias Tool [23] for RCTs and the Academy of Nutrition and Dietetics Quality Criteria Checklist [24] for other study designs. RCTs reporting on a subset of data not based on randomised status were assessed for study quality as a cohort design.

Where meta-analyses were possible, results of RCTs and observational studies were included as separate subgroups, using Review Manager (RevMan), Version 5.4.1 [25], with data expressed as mean difference (MD) with 95% confidence interval (CI). Where only medians with a measure of dispersion were available, these were converted to means and standard deviations (SD) using an online calculator (available at: http://www.math.hkbu.edu.hk/~tongt/papers/median2mean.html, accessed on 21 June 2021) [26,27]. Where mean and 95% CI were reported, these were converted to SD [23], and where groups were combined for meta-analyses, Cochrane methods were followed [23]. A random effects model was used where statistical heterogeneity was significant (I^2^ > 50%). Where studies have two or more intervention groups that fall into the designated categories, we report the combined means as calculated in Revman [25]. We have attempted to avoid including the same group of infants twice in the same meta-analysis, while striving to optimise precision by including relevant groups. Where it has not been possible to tease out the overlapping infants, we have not adjusted the *n* value but noted this in text, e.g., Huston 2018 [28], which includes some infants also reported in Huston 2014 [29], and noted if inclusion of this study changed the outcomes.

A ‘Summary of Findings’ table was prepared for each comparison using the GRADE system (GRADEpro GDT, 2015) [30]. RCTs with no limitations are considered high-quality evidence and observational studies as providing low-quality evidence. Studies can then be downgraded by one (for serious concern) or two (for very serious concerns) based on risk of bias, inconsistency, indirectness, imprecision, and publication bias. For each outcome, we report our certainty in the findings as very low, low, moderate, or high separately, according to study design (RCTs, observational).

To interpret the overall evidence for each outcome and comparison, we used the following terminology:

Clear effect/clear evidence of no effect: The certainty of evidence is moderate or above with a clinically important result from RCTs, ideally aligning with results from observational studies or moderate certainty evidence from observational studies and with reasonable numbers of events and/or participants.

Probably an effect/probably no effect: There is moderate certainty from either RCTs or observational studies, and point estimates may be different between the two study types with overlapping CIs but can be explained (e.g., through heterogeneity). There are large numbers of participants and studies.

Possible effect/possibly no effect: There is low/ moderate certainty with CIs, which may suggest a difference, although not reaching conventional statistical significance, or with a confidence interval, which indicates a trivial difference only.

Inconclusive: The certainty of evidence is very low to low, CIs are wide, and the number of participants and studies is low.

Where possible, the overall effect size (MD, 95% CI) has been reported. Figures showing forest plots for all outcomes are presented in the manuscript. For visual comparison, we have displayed a forest plot for comparisons that have only one study; however, we have computed a GRADE certainty rating only where there are two or more studies.

## 3. Results

The search and selection processes are presented in Supplementary Materials (Figure S1). Forty-four papers were identified for this review and included nine RCTs [15,19,20,21,31,32,33,34,35], one non-randomised intervention trial [36], three secondary analyses of RCTs [9,37,38], six interrupted time series studies [8,28,39,40,41,42], and 25 cohort studies [7,22,29,43,44,45,46,47,48,49,50,51,52,53,54,55,56,57,58,59,60,61,62,63,64]. Thirty-seven studies could be included in the meta-analyses [7,8,9,15,19,20,21,22,28,29,31,32,33,34,35,36,37,39,40,42,44,47,48,49,50,51,52,54,55,56,57,58,59,60,61,63,64].

For the four RCTs, we could directly compare data from randomised arms [31,32,33,34]. In five RCTs, we compared data to a non-randomised reference group, and consequently, these were assessed for study quality and treated as non-randomised trials [15,19,20,21,35]. Notably, for the RCT by Schanler 2005 [33], we also made comparisons between a randomised group and a non-randomised reference arm.

Table 1 provides details of the included studies. Individual summaries of findings tables for each comparison and outcome are presented in Supplementary Materials (Tables S2–S12), and a collated summary of findings table for all outcomes is presented in Table 2. Results for studies not included in a meta-analysis have been tabulated in Supplementary Materials (Table S1).

### 3.1. Risk of Bias and General Characteristics

Two of the RCTs [32,33] were rated as low risk of bias, one was assessed as moderate risk due to unclear sequence generation and allocation concealment [31], and another was considered high risk due to non-blinding of outcome assessors [34].

Of the non-randomised and observational studies, 28 were assessed as low risk of bias [7,8,9,15,19,20,21,22,35,38,39,40,41,42,43,45,47,49,50,52,55,57,58,59,60,61,63,64], 11 as moderate [28,29,36,37,44,46,48,51,54,56,62], and 1 as high [53] (Table 1).

There was some clinical heterogeneity in the time period over which growth was calculated, with starting points including birth, when birth weight was regained, weight nadir, attainment of 50% oral feeds, and study start; and endpoints including discharge, term corrected age, and study end (Table 1).

### 3.2. Weight Gain and Human Milk Feeding

Weight gain was reported in 41 studies [7,8,9,19,20,21,22,28,29,31,32,33,34,35,36,38,39,40,41,42,43,44,45,46,47,48,49,50,51,52,53,55,56,57,58,59,60,61,62,63,64]. Twenty-four studies reported g/kg/d, with 20 included in the meta-analysis [9,19,20,21,28,29,33,34,35,39,40,47,50,52,57,58,59,60,61,64] and four unable to be included [43,45,56,62]. Four studies reported weight gain in g/d [31,36,48,53], with three included in the meta-analysis [31,36,48]. Nineteen studies reported weight gain as change in z-scores with 14 included in meta-analyses [7,8,22,28,32,42,44,47,49,50,51,55,61,63] and five not included [38,41,43,46,62].

#### 3.2.1. Comparison 1: Exclusive Preterm Formula vs. Exclusive Human Milk

**Figure 1:**
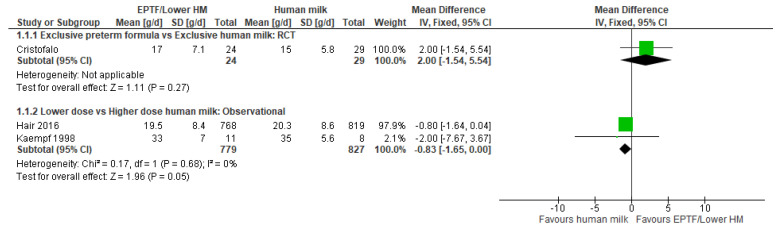
Forest plot of mean difference in weight gain (g/d) and human milk intake.

**Randomised Trials:** One small RCT [31] showed no difference in weight gain (g/d) between EPTF and EHM groups (MD 2.00, 95% CI −1.54 to 5.54, *n* = 53, Figure 1, moderate risk of bias, Table S2).

**Figure 2:**
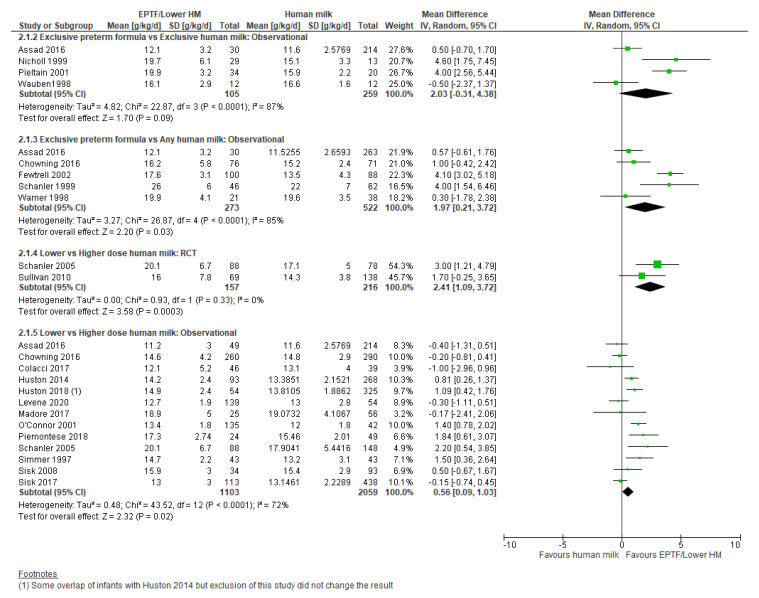
Forest plot of mean difference in weight gain (g/kg/day) and human milk intake.

**Observational studies:** Four studies reporting g/kg/d were included in the meta-analysis: [21,35,39,57]. There was no clear difference in the rate of weight gain between groups (MD 2.03, 95% CI −0.31 to 4.38, *n* = 364, I^2^ = 87%, Figure 2, very low certainty evidence, Table S3). The rate of weight gain was assessed across different time periods between studies, including from birth to discharge [39], full oral feeds tolerated for ≥5 days to discharge [21], enteral feed volume ≥150 mL/kg/d to nasogastric feeds no longer required [35], and full enteral feeding to discharge [57].

An additional two studies [45,53] were unable to be included in the meta-analysis. Carlson 1998 [45] reported weight gain (g/kg/d) according to different stages of the hospital admission and found higher weight gain with EPTF versus EHM fed infants over the time periods 15–35 days and 57 days to term (Table S1). In contrast, Manea 2016 [53] reported greater weight gain (g/d) in the EHM group during the first five weeks of life (Table S1).

**Figure 3:**
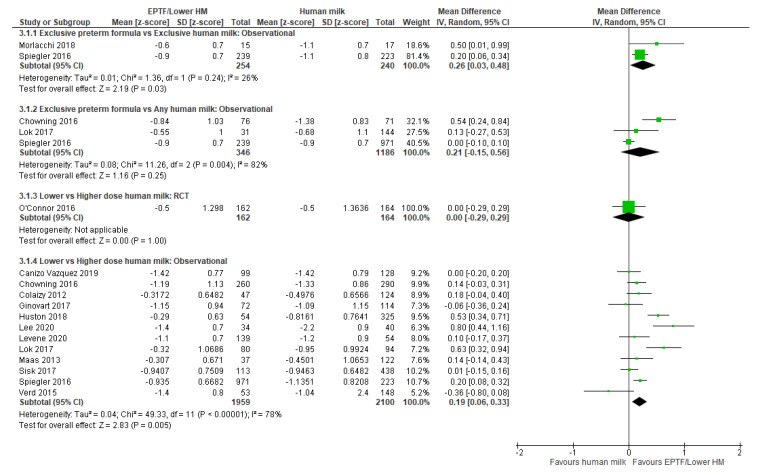
Forest plot of mean difference in change in weight z-scores and human milk intake.

Two studies reported change in z-scores [55,63] with EPTF-fed infants having a greater increase in z-scores than EHM-fed infants (MD 0.26, 95% CI 0.03 to 0.48, *n* = 494, I^2^ = 26%, Figure 3, low certainty evidence, Table S4).

**Overall:** The evidence for an effect of EPTF vs. EHM feeding on weight gain is inconclusive.

#### 3.2.2. Comparison 2: Exclusive Preterm Formula vs. Any Human Milk

**Randomised trials:** There were no RCTs reporting weight gain for this comparison.

**Observational studies:** Six studies reported weight gain in g/kg/d with five included in the meta-analysis [9,19,39,47,64]. EPTF-fed infants had a higher rate of weight gain (MD 1.97, 95% CI 0.21 to 3.72, *n* = 795, I^2^ = 85%, Figure 2, very low certainty evidence, Table S3). Heterogeneity may be explained by baseline difference in the study duration and varying intake of HM. Carlson 1998 [45] assessed weight gain over different time periods and showed higher weight gain (g/kg/d) in infants receiving EPTF compared with any HM over the time period 15–35 days and 57 days to term (Table S1).

Change in z-scores was reported in three studies [22,47,63] and all were included in a meta-analysis. There was no clear difference in change in z-score between infants receiving EPTF and any HM (MD 0.21, 95% CI 0.15 to 0.56, *n* = 1532, I^2^ = 82%, Figure 3, very low certainty evidence, Table S4). Heterogeneity may be due to the varying doses of HM (Table 1).

**Overall:** The evidence for an effect of EPTF vs. any HM feeding on weight gain is inconclusive.

#### 3.2.3. Comparison 3: Lower- vs. Higher-Dose Human Milk

**Randomised Trials:** Meta-analysis of two RCTs [33,34] showed a higher rate of weight gain (g/kg/d) in the lower-dose HM group (MD 2.41, 95%CI 1.09 to 3.72, *n* = 373, I^2^ = 0%, Figure 2, low certainty evidence, Table S3). O’Connor 2016 [32] showed no clear difference between lower- and higher-dose HM intake on change in weight z-score (MD 0.0, 95% CI −0.29 to 0.29, *n* = 326, Figure 3, low risk of bias, Table S4).

**Observational studies:** Two studies reported weight gain in g/d and were included in a meta-analysis [36,48] with a possible difference between lower and higher doses of HM (MD −0.83, 95% CI −1.65 to 0.00, *n* = 1606, I^2^ = 0%, Figure 1, low certainty evidence, Table S2).

Thirteen studies were included in the meta-analysis for weight gain (g/kg/d) for this comparison [20,28,29,33,39,40,47,50,52,58,59,60,61]. Lower doses of HM were associated with a higher rate of weight gain (g/kg/d) (MD 0.56, 95% CI 0.09 to 1.03, *n* = 3162, I^2^ = 72%, Figure 2, very low certainty evidence, Table S3). Heterogeneity is possibly due to differences in study design and varying dosage of HM. Also of note is that Sisk 2017 [61] used both a multi-nutrient and protein fortifier with the aim of providing a protein intake of 4 g/kg/d.

Two studies [43,62] reported weight gain as g/kg/d but were not included in the meta-analysis, as they did not directly compare groups [43] or did not report SDs [62] (Table S1). Brownell 2018 [43] showed that the mean growth rate decreased by 0.17 g/kg/d for every 10% increase in DHM intake but did not vary with PTF intake (using MOM as reference) (Table S1), whereas Soldateli 2020 [62] reported no difference in growth velocity (g/kg/d) across five categories of HM intake (*p* = 0.3) or between the lowest category (0–25% HM) and the highest category (100% HM) (*p* = 0.6) (Table S1).

Seventeen studies reported weight gain as change in z-scores during the hospital admission, and 12 of these were included in the meta-analysis [7,8,22,28,42,44,47,49,50,51,61,63]. Infants fed lower-dose HM had a greater increase in z-score change than those fed higher-dose HM (MD 0.19, 95% CI 0.06 to 0.33, *n* = 4059, I^2^ = 78%, Figure 3, very low certainty evidence, Table S4). Heterogeneity is possibly due to difference in doses of HM and differences in type of fortifier used. Five studies [38,41,43,46,62] were not included in a meta-analysis. Soldateli 2020 [62] showed no difference in change in weight z-score across five categories of HM intake (*p* = 0.7) or between the lowest category (0–25% HM) and the highest category (100% HM) (*p* = 0.2) (Table S1). The remaining four studies [38,41,43,46] all reported a greater increase in weight z-score associated with lower doses of HM (Table S1).

**Overall:** There is a possible effect that lower doses of HM compared with higher doses of HM improve weight gain during the hospital admission.

### 3.3. Head Circumference Gain and Human Milk Feeding

Thirty studies reported HC gain. Nineteen studies reported HC growth in cm/wk [9,19,20,21,28,29,31,33,34,43,47,48,52,56,57,59,61,62,64], and 16 reported change in z-scores [8,22,28,32,38,41,43,44,46,47,50,51,55,61,62,64].

#### 3.3.1. Comparison 1: Exclusive Preterm Formula vs. Exclusive Human Milk

**Figure 4:**
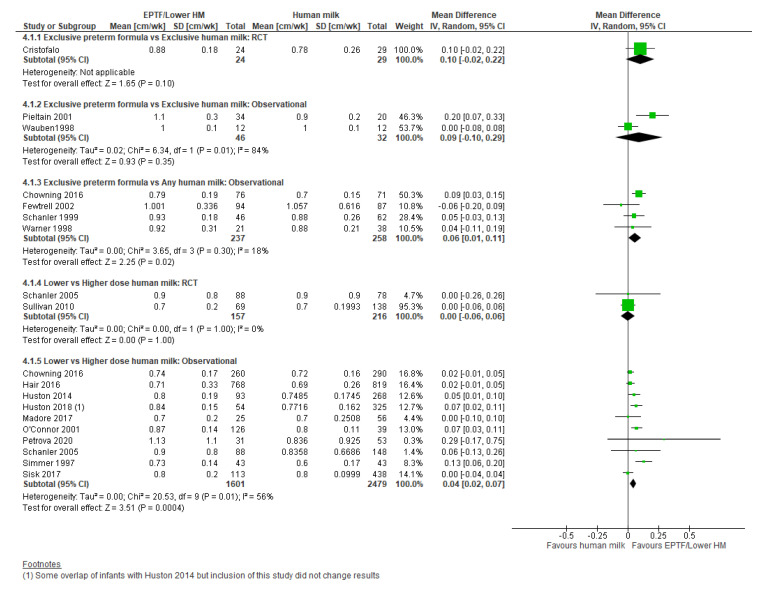
Forest plot of mean difference in change in head circumference gain (cm/wk) and human milk intake.

**Randomised trials:** There was no clear difference in HC growth (cm/wk) in the one RCT [31] reporting this comparison (MD 0.10, 95% CI −0.02 to 0.22, *n* = 53, Figure 4, moderate risk of bias, Table S5).

**Observational studies:** Meta-analysis of two studies [21,57] showed no clear difference in HC gain (cm/wk) (MD 0.09, 95% CI −0.10 to 0.29, *n* = 78, I^2^ = 84%, Figure 4, very low certainty evidence, Table S5). Heterogeneity is likely due to baseline differences in population and study design.

**Figure 5:**
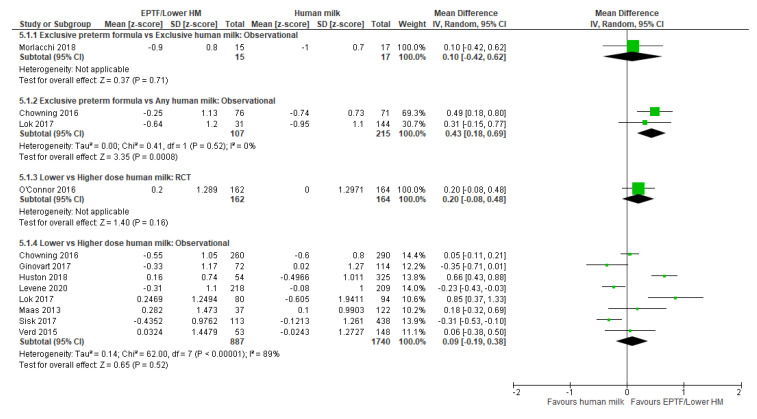
Forest plot of mean difference in change in head circumference z-scores and human milk intake.

One study reported change in HC z-scores [55] and showed no clear difference between the EPTF and EHM fed groups (MD 0.10, 95% CI −0.42 to 0.62, *n* = 32, Figure 5, low risk of bias, Table S6).

**Overall:** The evidence for an effect of EPTF vs. EHM feeding on HC growth is inconclusive.

#### 3.3.2. Comparison 2: Exclusive Preterm Formula vs. Any Human Milk

**Randomised trials:** There were no RCTs identified reporting HC gain for this comparison.

**Observational studies:** Four studies [9,19,47,64] reported HC gain (cm/wk) in infants fed EPTF compared with any HM. On meta-analysis, EPTF-fed infants had a higher rate of HC gain (MD 0.06, 95% CI 0.01 to 0.11, *n* = 495, I^2^ = 18%, Figure 4, low certainty evidence, Table S5).

Two studies [22,47] reported change in HC z-score, and on meta-analysis, there was a greater increase in HC z-scores in the infants fed EPTF (MD 0.43, 95% CI 0.18 to 0.69, *n* = 322, I^2^ = 0%, Figure 5, low certainty evidence, Table S6).

**Overall:** There is a possible effect that feeding EPTF compared with any HM is associated with small increases in HC gain during the hospital admission.

#### 3.3.3. Comparison 3: Lower- vs. Higher-Dose Human Milk

**Randomised trials:** There was no clear difference in HC growth (cm/wk) in the meta-analysis of the two RCTs reporting this outcome [33,34] (MD 0.00, 95% CI −0.06 to 0.06, *n* = 373, I^2^ = 0%, Figure 4, moderate certainty evidence, Table S5). One RCT [32] reported change in HC z-score and showed no difference between groups (MD 0.20, 95% CI −0.08 to 0.48, *n* = 326, Figure 5, low risk of bias, Table S6).

**Observational studies:** Twelve studies reported HC growth in cm/wk with 10 included in the meta-analysis [20,28,29,33,47,48,52,56,59,61] showing greater HC gain associated with lower-dose HM (MD 0.04, 95% CI 0.02 to 0.07, *n* = 4080, I^2^ = 56%, Figure 4, very low certainty evidence, Table S5).

Two studies could not be included in the meta-analysis [43,62]. Brownell 2018 [43] showed that, in reference to MOM, increased DHM intake was associated with decreased HC growth (cm/wk), but PTF was not (Table S1). Soldateli 2020 [62] reported no difference in HC growth (cm/wk) across five categories of HM intake (*p* = 0.4) or between the lowest category (0–25% HM) and the highest category (100% HM) (*p* = 0.1) (Table S1).

Thirteen studies reported change in HC z-scores, with eight included in the meta-analysis [8,22,28,42,47,50,51,61]. There was no clear difference in change in HC z-score between lower and higher-dose HM-fed infants (MD 0.09, 95% CI −0.19 to 0.38, *n* = 2627, I^2^ = 89%, Figure 5, very low certainty evidence, Table S6). Heterogeneity is likely due to difference in proportion of HM and PTF dosage.

Five studies reported change in HC z-scores but could not be included in the meta-analysis [38,41,43,46,62]. Three studies [38,41,43] compared the difference between a reference, either MOM [41,43] or EHM [38], and found no relationship between formula intake and HC, although Brownell 2018 [43] also found that increased DHM intake was significantly associated with decreased change in HC z-scores (Table S1). Castellano Yanez 2019 [46] reported a greater increase in HC z-scores with lower-dose HM (Table S1). However, Soldateli 2020 [62] reported no difference in change in HC z-score across five categories of HM intake (*p* = 0.8) or between the lowest category (0–25% HM) and the highest category (100% HM) (*p* = 0.2) (Table S1).

**Overall:** There is possibly no effect of lower vs. higher doses of HM feeding on HC growth.

### 3.4. Length Gain and Human Milk Feeding

Twenty-five studies reported length gain in a variety of ways: 15 as cm/wk [9,20,21,28,29,31,33,34,37,43,52,57,59,61,64], 11 as change in z-score [28,32,38,41,42,43,44,46,55,61,62], and two studies reported lower leg growth [35,36].

#### 3.4.1. Comparison 1: Exclusive Preterm Formula vs. Exclusive Human Milk

**Figure 6:**
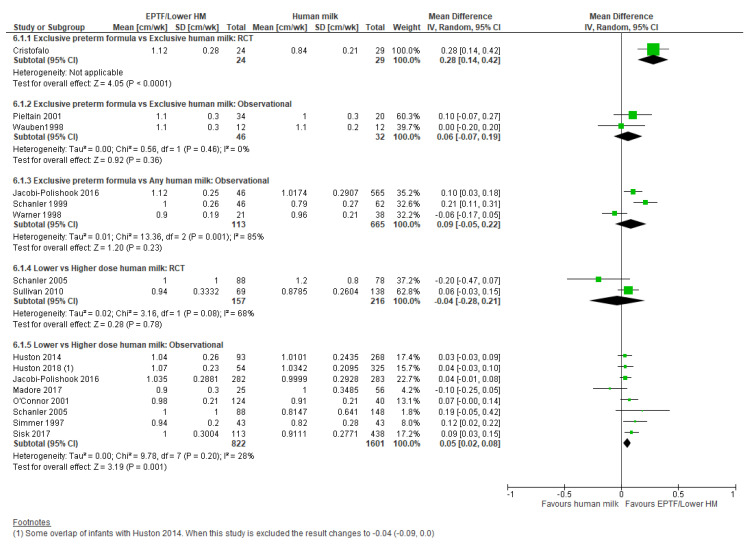
Forest plot of mean difference in change in length gain (cm/wk) and human milk intake.

**Randomised trials:** One small RCT [31] reported the effect of EPTF feeding compared with EHM feeding on linear growth and found a higher length gain (cm/wk) with EPTF (MD 0.28, 95% CI 0.14 to 0.42, *n* = 53, Figure 6, moderate risk of bias, Table S7).

**Observational studies:** The impact of EPTF vs. EHM feeding on linear growth (cm/wk) was addressed in two studies [21,57]. On meta-analysis, there was no clear difference in linear growth between groups (MD 0.06, 95% CI −0.07 to 0.19, *n* = 78, I^2^ = 0%, Figure 6, very low certainty evidence, Table S7).

Nicholl 1999 [35] investigated the effect of feeding variation on lower leg length gain from the time of enteral feeds reaching ≥150 mL/kg/d until nasogastric feeds ceased. There was no difference between groups (Table S1).

**Figure 7:**
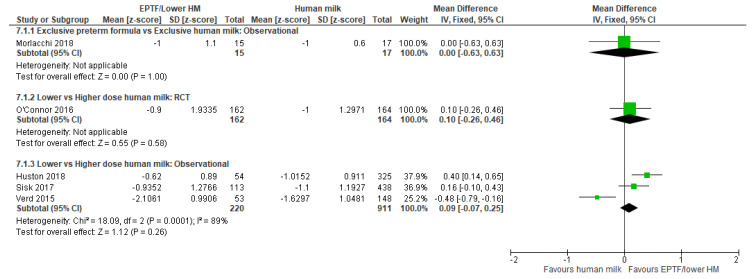
Forest plot of mean difference in change in length z-scores and human milk intake.

Change in length z-scores were reported in one study [55] with no clear difference detected (MD 0.00, 95%CI −0.63 to 0.63, *n* = 32, Figure 7, low risk of bias, Table S8).

**Overall:** The evidence for an effect of feeding EPTF vs. EHM on length gain is inconclusive.

#### 3.4.2. Comparison 2: Exclusive Preterm Formula vs. Any Human Milk

**Randomised trials:** There were no RCTs identified reporting length gain for this comparison.

**Observational studies:** Three studies [9,37,64] reported length gain (cm/wk) for this comparison and were included in the meta-analysis. No clear difference in length gain was shown between groups (MD 0.09, 95% CI −0.05 to 0.22, *n* = 778, I^2^ = 85%, Figure 6, very low certainty evidence, Table S7). Heterogeneity may be explained by different dosages of HM.

**Overall:** The evidence for an effect of feeding EPTF vs. any HM on length gain is inconclusive.

#### 3.4.3. Comparison 3: Lower- vs. Higher-Dose Human Milk

**Randomised trials**: No clear difference was shown in the meta-analysis of two RCTs [33,34] reporting length gain (cm/wk) (MD −0.04, 95% CI −0.28 to 0.21, *n* = 373, I^2^ = 68%, Figure 6, low certainty evidence, Table S7). One RCT [32] reported change in length z-score and showed no clear difference between lower and higher-dose HM (MD 0.10, 95% CI −0.26 to 0.46, *n* = 326, Figure 7, low risk of bias, Table S8).

**Observational studies**: Nine studies reported length gain (cm/wk) with eight included in the meta-analysis [20,28,29,33,37,52,59,61]. Infants fed lower-dose HM compared with a higher dose showed a slightly higher length gain (MD 0.05, 95% CI 0.02 to 0.08, *n* = 2423, I^2^ = 24%, Figure 6, low certainty evidence, Table S7).

Two studies [36,43] were unable to be included in the meta-analysis. Brownell 2018 [43] reported length velocity using MOM as reference, and neither the proportion of DHM nor PTF intake were associated with length gain (Table S1). Kaempf 1998 [36] reported the effect of fortified BM (>80% fortified BM) vs. PTF (>80% PTF) feeding on lower leg gain (mm/d) and found no difference between groups (Table S1).

Eight studies reported length as change in z-scores, with three included in the meta-analysis [28,42,61]. There was no clear difference in change in length z-scores between groups (MD 0.09, 95% CI −0.07 to 0.25, *n* = 1131, I^2^ = 89%, Figure 7, very low certainty evidence, Table S8). For the remaining five studies, the results were variable, with two studies [41,46] showing a greater increase in length z-scores with lower-dose HM and two studies showing no difference in change in length z-score [38,62], whereas Brownell 2018 reported that only PTF intake proportion was associated with decreased change in length z scores (Table S1).

**Overall:** There is possibly no effect of lower vs. higher doses of HM feeding on length gain.

### 3.5. Body Composition and Human Milk Feeding

Body composition was reported in eight observational studies [15,37,38,54,55,57,58,65] and measured using different techniques: two studies used dual-energy X-ray absorptiometry [57,65]; two used air displacement plethysmography [55,58]; two used either bioelectrical impedance analysis [15] or bioelectrical impedance spectroscopy [54]; one study used full body magnetic resonance imaging [38]; and one reported Body Mass Index (BMI) [37].

#### 3.5.1. Comparison 1: Exclusive Preterm Formula vs. Exclusive Human Milk

**Randomised trials:** There were no RCTs identified for this comparison.

**Figure 8:**
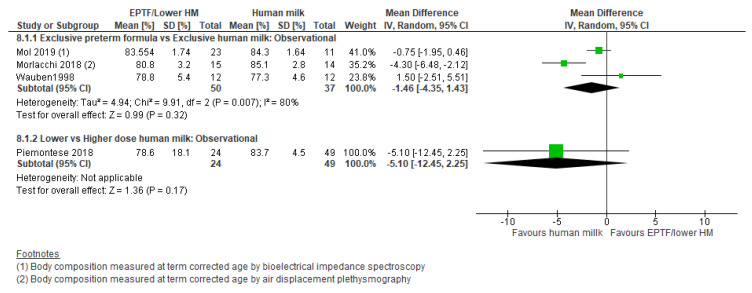
Forest plot of mean difference in change in % fat-free mass and human milk intake.

**Figure 9:**
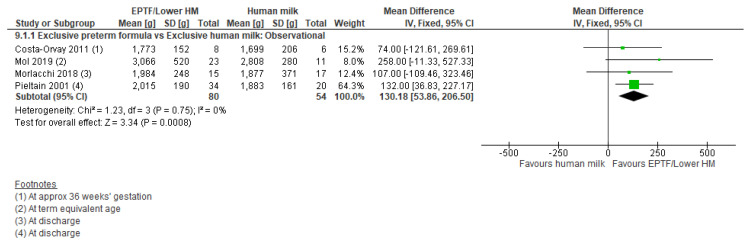
Forest plot of mean difference in change in fat-free mass (g) and human milk intake.

**Observational studies:** Three studies [21,54,55] reported % fat-free mass and showed no clear difference between groups (MD −1.46, 95% CI −4.35 to 1.43, *n* = 87, I^2^ = 80%, Figure 8, very low certainty evidence, Table S9). Four studies reported fat-free mass (g) [15,54,55,57] and showed an increase with EPTF (MD 130.18, 95% CI 53.86 to 206.50, *n* = 134, I^2^ = 0%, Figure 9, very low certainty evidence, Table S10).

**Figure 10:**
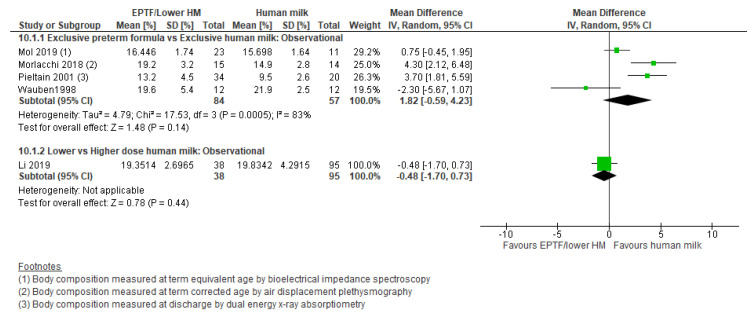
Forest plot of mean difference in change in % fat mass and human milk intake.

**Figure 11:**
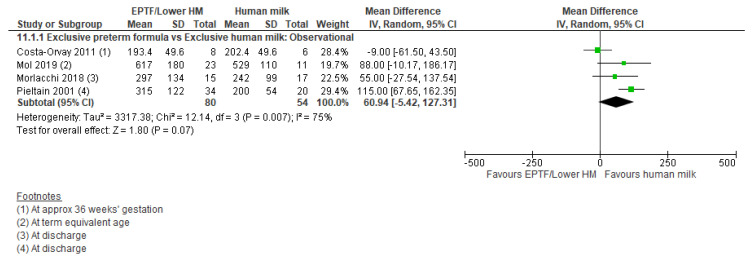
Forest plot of mean difference in change in fat mass (g) and human milk intake.

Four studies [54,55,57,65] reported % fat mass and showed no clear difference between groups (MD 1.82, 95% CI −0.59 to 4.23, *n* = 141, I^2^ = 83%, Figure 10, very low certainty evidence, Table S11). Four studies [15,54,55,57] reported fat mass (g) and showed no clear difference between groups (MD 60.94, 95% CI −5.42 to 127.31, *n* = 134, I^2^ = 75%, Figure 11, very low certainty evidence, Table S12).

**Overall:** The evidence for an effect of feeding EPTF vs. EHM on fat and fat-free mass is inconclusive.

#### 3.5.2. Comparison 2: Any Human Milk vs. Exclusive Preterm Formula

There were no studies identified for this comparison.

#### 3.5.3. Comparison 3: Lower- vs. Higher-Dose Human Milk Intake

Piemontese 2018 [58] reported % fat-free mass and showed no clear difference between groups (MD −5.10, 95% CI −12.45 to 2.25, *n* = 73, Figure 8, low risk of bias, Table S9). Li 2019 [38] reported fat-free mass (g) with the predominantly formula-fed group having greater fat-free mass than EHM (MD 257.4, 95% CI 139.1 to 357.7, *n* = 95, *p* < 0.01, Table S1).

Li 2019 [38] reported % fat mass and showed no clear difference between groups (MD −0.48, 95% CI −1.70 to 0.73, *n* = 133, Figure 10, low risk of bias, Table S11). Li 2019 [38] also reported fat mass (g) with no clear difference between EHM and predominantly HM or predominantly formula (Table S1).

Jacobi-Polishook 2016 [37] reported BMI gain from birth to discharge (kg/m^2^/wk) with similar BMI gain across quartile of HM intake (Table S1).

**Overall:** The evidence for an effect of feeding lower vs. higher-dose HM on fat and fat-free mass is inconclusive.

## 4. Discussion

### 4.1. Summary of Main Results

Forty-four studies were included in this review, of which 37 could be included in meta-analyses (4 RCTs with 866 infants and 33 observational studies with 9097 infants). Seven studies with 1917 infants were synthesised narratively. Overall, there was inconclusive evidence to draw reliable conclusions about the effect of HM feeding on growth outcomes in very low birth weight infants. There is a possible effect that lower doses of HM compared with higher doses of HM improve weight gain during the hospital admission; the overall quality of the evidence was low to very low for most outcomes; thus, our confidence in the results is limited. The majority of studies included in this review were categorised in the lower versus higher-dose HM comparison, with insufficient evidence to reliably assess the effect an exclusive HM diet versus EPTF on any outcomes.

We included measures of body composition in our review to examine the possible effects of HM feeding on quality of growth. Few studies reported these outcomes. The available evidence was poor quality but did suggest that the proportion of fat mass (%) at term corrected age was significantly lower in EHM-fed vs. EPTF-fed infants, a positive effect of HM, as lower fat mass at term corrected age better aligns with infants born at term [66]. These findings warrant confirmation in further large-scale studies and reiterate the need for inclusion of measures of not only growth velocity but also quality (e.g., fat-free mass) in studies examining long-term outcomes of preterm infants.

### 4.2. Findings from Other Reviews

While there are a large number of studies reporting on growth among preterm infants fed with fortified HM, there are few systematic reviews published in this area. The available studies are largely observational; a recent Cochrane review examining the effects of formula vs. maternal milk feeding for preterm infants failed to identify any RCTs that met their criteria [10]. Another Cochrane review by Quigley and colleagues compared formula and donor breast milk for feeding preterm infants [11] and included a subgroup analysis of fortified donor HM with preterm formula, which is the closest match to our review. They also found an effect of higher growth rates in favour of preterm formula for all measures. The three studies that their review comprises [31,32,33] are also included in our meta-analysis.

Our review provides a more complete summary of the evidence concerning HM intake and growth outcomes as we included non-randomised study designs and did not apply any restrictions regarding the source of milk (e.g., MOM or DHM). Nevertheless, our findings regarding weight gain were similar to the effect size reported by Quigley et al. [11] (MD 2.37, 95% CI 1.09 to 3.65) g/kg/d), suggesting slower weight gain with cumulative HM intake. However, we rated the overall evidence as inconclusive, as many of the included studies were small and thus underpowered and/or had major methodological limitations.

Unlike Quigley et al. [11], we did not find consistent results for length gain, either when reported as cm/week or change in z-score; however, this was sparsely reported across different comparison groups, and thus should be interpreted with caution. We did identify a possible effect of increased HC gain with EPTF vs. any HM feeds, based on data predominantly from observational studies, which is also inconsistent with the findings of the Quigley review. However, findings were not consistent across HM exposure groups. For the comparison of low versus higher-dose HM, we concluded that there was possibly no effect of dose of HM on HC gain, based on moderate certainty of evidence generated from RCTs and trivial differences identified in the observational studies reporting this outcome. Notably, across the comparisons where head growth was reported, the pooled effect size was less than 0.1 cm/week. When calculated over a 12-week admission, this equates to a difference of just under 1 cm in head circumference: roughly equivalent to a centile space on intrauterine growth curves.

Previous studies have shown that faster head growth before term and post-discharge is associated with small improvements in longer-term neurodevelopmental outcomes [16,67]. This has led to an increased focus on early intensive parenteral and enteral nutritional support in preterm infants. However, Rozé and colleagues analysed data from two recent large cohort studies of nearly 3000 very preterm infants and identified inconsistencies in the relationship between early growth and developmental outcomes in breastfed infants, in what the authors term the ‘apparent breastfeeding paradox’ [68]. They found breastfeeding at discharge was associated with a 1.5–2.5 increase in the odds of losing one weight z-score during hospitalisation but with an increased odds of having an HC z-score higher than 0.5 at 2 years and a decreased risk of suboptimal neurodevelopment at 2 and 5 years of age, respectively [68]. Although not an outcome of this review, a previous meta-analysis found there is inconclusive evidence regarding a possible effect of HM feedings on cognitive and motor development in VLBW infants [3].

### 4.3. Implications for Clinical Practice and Research

HM should continue to be promoted as the optimal source of nutrition for all infants, given the known benefits beyond infant growth. However, further high-quality research is needed to delineate the complex relationships between infant feeding practices, weight gain, body composition, and later neurodevelopment in VLBW infants. In particular, clarity is needed regarding the optimal ways to feed expressed HM to VLBW infants in the early weeks and months after birth. The inconclusive findings identified in this review are likely heavily influenced by differences in feeding management between studies, given the variety of settings in which the studies were conducted. This includes practices regarding fortification of expressed breast milk, including individual versus standardised fortification regimens, as well as changes in the makeup of commercially available fortifiers.

The protein concentration of fortifiers has generally increased over time, and there is evidence of small increases in weight in hospital with the use of higher protein versus lower protein concentration fortifiers [69]. While we selected studies that used contemporary feeding approaches such as fortified HM, the level of fortification varied considerably and was often not reported. The source of the fortifier also varied, with some recent studies using a human milk-derived fortifier rather than the more commonly available commercial bovine milk-derived fortifiers. Recent meta-analyses indicate potential clinical benefits associated with the use of a human milk-derived vs. bovine milk-derived fortifier, although only one study reported on growth outcomes and found no difference [70,71].

In addition, there was significant variation in practices surrounding the use of DHM between studies. This has the potential to influence growth outcomes, as the protein concentration of DHM is highly variable and influenced by the lactational stage of the donors [72]. The pasteurisation and storage practices concerning DHM are also known to affect the concentration of bioactive proteins and other components of HM [73].

Future studies should be sufficiently large enough to examine the effects of an exclusive HM diet, as well as potential threshold and dose–response relationships, on growth and longer-term developmental outcomes in preterm infants. Studies must include detailed description of feeding management practices to permit a more accurate estimate of protein and energy intakes among participants. This will generate evidence to better define the cumulative effects of HM feeds that will help inform the optimal feeding strategies in the neonatal unit, particularly while breastfeeding is being established. In addition, detailed collection and reporting based on the source of HM (mother’s own or donor) are needed to clarify any specific impact of DHM feeds on growth and neurodevelopmental outcomes.

### 4.4. Strengths and Limitations

We have used robust methods to systematically search, synthesise, and critique the evidence. At least two reviewers independently abstracted data and assessed the quality and certainty of evidence using established tools. However, it is nevertheless possible that studies were not identified.

There are several limitations. There is considerable heterogeneity, both clinical and statistical, in the included studies. This in part reflects our deliberate approach to allow a range of HM exposures and a broad range of outcome measures. For example, there was some variation in the volume of HM intake among studies included in the ‘any HM vs. PTF’ and ‘high- vs. low-dose’ comparisons, and in some cases, the volume was not specifically reported. In addition, growth was measured over varying time points, and different protocols for length, head circumference, and body composition measurement between studies may have led to measurement errors and thus heterogeneity in the meta-analyses.

Differences in clinical management, including feeding practices, as described earlier, are another significant source of heterogeneity. Teasing out the effects of fortification and DHM was not possible in this review, as often these practices were poorly described in individual studies.

Most of the evidence reviewed is from observational studies, including five RCTs where comparisons relevant to the review included a non-randomised arm and therefore carry an increased risk of bias. We deliberately included both RCTs and observational studies in this review to provide a comprehensive synthesis of the available evidence, and we have accounted for study design by using the GRADE system to decide the certainty of evidence. Nevertheless, many of the studies had small sample sizes and included growth as secondary outcomes. Interpretation of the evidence is complex, particularly in comparisons with data from both RCTs and observational studies, where the effect is not in alignment. For this reason, we took a conservative approach and defined our interpretation of the evidence a priori to ensure consistency.

## 5. Conclusions

Although we identified a large number of studies involving over 10,000 VLBW infants, we have found much of the evidence for the association between growth outcomes and HM intake to be inconclusive, largely due to the quality of the evidence. While the meta-analysis demonstrated possible effects of increased weight gain among infants fed lower doses of HM and increased head circumference gain among those fed EPTF vs. any HM, the certainty of the body of the available evidence was very low to low. The effect sizes were also small; thus, the clinical significance of these differences is unclear. Carefully designed studies that assess dose-dependent effects and account for the source of milk and specific protein and energy fortification practices are needed to inform optimal HM feeding strategies in the neonatal unit.

## Figures and Tables

**Table 1:** Characteristics of included studies.

Study Country	Design	Participants BW, g; GA, wk; *n*	Intervention (Proportion of HM % Unless Stated Elsewhere)	Comparisons for This Review C1: EHM vs. EPTF C2: Any HM vs. EPTF C3: High- vs. Low-Dose HM	Study Duration	Outcomes	Risk of Bias
**Randomised Controlled Trials**							
Cristofalo (2013) [31] USA and Australia	RCT	53 infants Gp1: 996 ± 152; 27.7 ± 1.5; 29 Gp2: 983 ± 207; 27.5 ± 2.4; 24	Gp1: EHM (HMDF) (100%) Gp2: EPTF (BovF) (0%)	C1: Gp1 vs. Gp2	SS: Initiation of enteral nutrition SE: Earliest of 91 d of age; DC; or 50% oral feedings	Growth (from regained BW to SE): wt gain (g/d), HC gain (cm/wk), Length gain (cm/wk)	Moderate (sequence generation and allocation concealment unclear)
O’Connor 2016 [32] Canada	RCT	363 infants Gp1: 995 ± 273; 27.5 ± 2.4; 181 Gp2: 996 ± 272; 27.8 ± 2.7; 182	Gp1: EHM (MOM + DHM) (100%; MOM 58.4% (13.6, 96.0)) Gp2: Mixed (MOM + PTF) (MOM 63.3% (9.6, 97.2))	C3: Gp1 vs. Gp2	SS: Start enteral feeds SE: 90 d or DC	Growth (during intervention): change in wt, HC and length z-score	Low
Schanler 2005 [33] USA	RCT (randomised arms) Cohort (non-randomised arm)	243 infants Gp1: 947 ± 233; 27 ± 2; 81 Gp2: 957 ± 267; 27 ± 2; 92 Gp3: 999 ± 259; 27 ±2; 70	Gp1: EHM (MOM + DHM) (100%) Gp2: Mixed (MOM + PTF) (NR) Gp3: EHM (MOM) (100%) (reference, not randomised)	C3 (RCT): Gp1 vs. Gp2 C3 (non-RCT): Gp 1+ Gp3 vs. Gp2	SS: 4 d after birth SE: 90 d of age or DC	Growth (during study): wt gain (g/kg/d), HC gain (cm/wk), Length gain (cm/wk)	Low (sequence generation unclear)
Sullivan 2010 [34] USA and Austria	RCT	207 infants Gp1: 945 ± 202; 27.2 ± 2.2; 67 Gp2: 909 ± 193; 27.1 ± 2.3; 71 Gp3: 922 ± 197; 27.3 ± 2.0; 69	Gp1: EHM (MOM + DHM + HMDF) (100%; MOM 73% (16, 82)) (fortified at 100 mL/kg/d) Gp2: EHM (MOM + DHM + HMDF) (100%; MOM 70% (18, 80)) (fortified at 40 mL/kg/d) Gp3: Mixed (MOM + PTF + BovF) (NR; MOM 82% (38, 100)) (fortified at 100 mmL/kg/d)	C3: Gp1 + 2 vs. Gp 3	SS: Start of enteral nutrition SE: Earliest of 91 d of age, DC, 50% oral feedings	Growth (from SS to SE): mean wt gain (g/kg/day), HC and length gain (cm/wk); HC and length gain reported as median, IQR, and converted to mean, SD.	High (no blinding of caregivers who likely measured growth)
**Observational Studies**							
Assad 2016 [39] USA	Interrupted time series	293 infants BW range: 490 to 1700 GA Gp1: 27.7 ± 2.7; 87 Gp2: 28.3 ± 2.8; 127 Gp3: 27.6 ± 2.8; 49 Gp4: 29.8 ± 2.5; 30	Gp1: EHM (MOM + DHM + HMDF) (100%) Gp2: EHM (MOM + BovF) (100%) Gp3: Mixed (MOM + BovF + PTF) (NR) Gp4: EPTF (0%)	C1: Gp1 + Gp2 vs. Gp4 C2: Gp1 + Gp2 + Gp3 vs. Gp4 C3: Gp1 + Gp2 vs. G3	NR	Growth (from birth to DC): wt gain (g/kg/d)	Low
Brownell 2018 [43] USA	Cohort	314 infants Whole cohort: 1233 ± 373; 29.5 ± 2.9; 314	10% incremental exposure to MOM, DHM, and PTF	C3: synthesised narratively	SS: enteral feedings start SE: 36 wk PMA or DC	Growth (from birth to 36 wk PMA): wt gain (g/kg/day), HC gain (cm/wk), length gain (cm/wk), change in wt, length, and HC z-scores	Low
Canizo Vazquez 2019 [44] Spain	Cohort	227 infants Gp 1: 1283 ± 393; 29.5 ± 2.3; 99 Gp 2: 1197 ± 370; 29.1 ± 2.3; 128	Gp1: MOM, PTF Gp2: MOM, DHM	C3: Gp2 vs. Gp1	Hospital stay	Growth (from birth to discharge): change in wt z-score; length, and HC z-score at SE	Moderate (Proportion of HM not reported)
Carlson 1998 [45] USA	Cohort	51 infants Whole cohort: 899 ± 205; 27.1 ± 1.9; 51	Gp1: EHM (MOM) (100%) Gp2: Mixed (MOM + PTF) (NR) Gp3: EPTF (0%)	C1, C2, C3: synthesised narratively	NR	Growth (from birth to DC): wt gain (g/kg/day), measured in time periods: 0–14 d, 15–35 d, 36–56 d, 57 d to TCA	Low
Castellano Yanez 2019 [46] Spain	Cohort	130 infants Gp 1: 1430 ± 262; 31.2 (30.1, 31.7); 52 Gp 2: 1343 ± 233; 31.5 (30.0, 32.7); 78	Gp1: MOM, PTF Gp2: MOM, DHM	C3: synthesised narratively	Hospital stay	Growth (from birth to DC): change in wt, length, and HC z-scores—difference between groups	Moderate (some differences between Gps)
Chowning 2016 [47] USA	Cohort	550 infants Gp1: 1030 ± 290; 28.1 ± 2.7; 260 Gp2: 1080 ± 280; 28.6 ± 2.5; 290 Gp3: 1150 ± 290; 29.3 ± 2.8; 76 Gp4: 1160 ± 240; 29.1 ± 2.4; 71	Gp1: <50% d received HM Gp2: ≥50% d received HM Separate analysis done for extremes of intake Gp3: 0% d received HM Gp4: ≥90% d received HM	C2: Gp4 vs. Gp3 C3: Gp2 vs. Gp1	Hospital stay	Growth: wt gain (from regained BW to DC, g/kg/d), HC gain (from birth to DC, cm/wk), Change in wt and HC z-score (from birth to DC)	Low
Colacci 2017 [40] USA	Interrupted time series	85 infants Gp1: 783 ± 143; 26.0 ± 1.9; 39 Gp2: 770 ± 137; 26.0 ± 1.9; 46	Gp1: EHM (MOM + DHM + HMDF) (100%) Gp2: Mixed (MOM + PTF + BovF) (83% of feeding as formula)	C3: Gp1 vs. Gp2	SS: birth SE: at least for 4 wk and wt ≥1500 g or 34 wk PMA (whichever occur first)	Growth (from birth to DC): wt gain (g/kg/d)	Low
Colaizy 2012 [7] USA	Cohort	171 infants Gp1: 1083 (778, 1184); 28.4 (25.4, 29.6); 17 Gp2: 861 (736, 1091); 26.89(25.4, 29.0); 30 Gp3: 848 (717, 1011); 26.6(25.7, 28.5); 36 Gp4: 880 (719, 1052); 27(25.6, 28.8); 88	Gp1: <25% HM (MOM + DHM + PTF) Gp2: 25–50% HM (MOM + DHM + PTF) Gp3: 51–75% HM (MOM + DHM + PTF) Gp4: >75% HM (MOM + DHM + PTF)	C3: Gp1 + Gp2 vs. Gp3 + Gp4	Hospital stay	Growth (from birth to DC): change in wt z-score (reported as median, IQR and converted to mean, SD)	Low
Costa-Orvay 2011 [15] Spain	RCT with non-randomised reference group	38 infants Gp1: 1138 ± 173; 29.0 ± 1.7; 6 Gp2: 1196 ± 243; 29.6 ± 1.6; 8 Gp3: 1220 ± 221; 30.2 ± 1.4; 12 Gp4: 1313 ± 336; 29.8 ± 1.7; 12	Gp1: EHM (MOM + BovF) (100%), (reference, not randomised) Gp2: EPTF (0%) Gp3: EPTF (high energy and protein formula) (0%) Gp4: EPTF (high energy and protein formula) (0%)	C1: Gp1 vs. Gp2	Intervention: 4 wks from regain BW	Body composition (BIA) at SE: FM (g), FFM (g)	Low
Fewtrell 2002 [19] United Kingdom	RCT with non-randomised reference group	283 infants Gp1: 1353 ± 274; 30.3 ± 2.4; 100 Gp2: 1336 ± 284; 30.4 ± 2.3; 95 Gp3: 1395 ± 262; 30.3 ± 2.0; 88	Gp1: Control PTF (0%) Gp2: LCPUFA-supplemented formula (0%) Gp3: MOM (NR) (reference, not randomised)	C2: Gp3 vs. Gp1	SS: 10 d of age SE: 18 m CA	Growth (from birth to DC): wt gain (g/kg/d), HC gain (cm/wk)	Low
Ginovart 2017 [8] Spain	Interrupted time series	182 infants Gp1: 1108 ± 273; 29^+4^ ± 2^+6^; 72 Gp2: 1078 ± 289; 29^+1^ ± 2^+6^; 114	Gp1: Any PTF (PTF + MOM) (NR) Gp2: EHM (MOM + DHM + BovF) (100%)	C3: Gp2 vs. Gp1	Hospital stay	Growth (from birth to DC): change in wt and HC z-score	Low
Hair 2016 [48] USA	Cohort	1587 infants Gp1: 823 ± 205; 26.4 ± 2.3; 768 Gp2: 844 ± 210; 26.5 ± 2.5; 819	Gp1: MOM + BovF + PTF (NR) Gp2: EHM (MOM+ DHM + HMDF) (100%)	C3: Gp2 vs. Gp1	SS: NR SE: Varied between sites: 34 wk PMA, 60 d of age, at 1500 g or 34 wk PMA, 32 wk PMA	Growth (time frame for measures NR): wt gain (g/d), HC gain (cm/wk)	Moderate (some differences between Gps)
Hoban, 2019 [41] USA	Interrupted time series	321 infants Gp1: Median (IQR) 1050 (750, 1220); 2707 (26.1, 29.4); 160 Gp2: 1000 (800, 1180); 27.7 (25.9, 29.3); 161	Gp1: Pre-DM era (97% (35, 100) Gp2: DM era (100%)	C3: Gp 2 vs. Gp1 Synthesised narratively	Feeding collected 1st 28 days of life	Growth (birth to DC): change wt, length, and HC z-scores	Low
Huston 2014 [29] USA	Cohort	361 infants Gp1: 1177 ± 222; 29.1 ± 1.8; 93 Gp2: 1104 ± 262; 28.1 ± 2.2; 224 Gp3: 919 ± 269; 26.7 ± 2.4; 44	Gp1: EPTF or MOM + BovF + PTF for >48 h (NR) Gp2: MOM + DHM + BovF (100%) Gp3: EHM (MOM + DHM + HMDF) (100%)	C3: Gp2 + Gp3 vs. Gp1	NR	Growth (during hospital stay): wt gain (g/kg/d), HC gain (cm/wk), length gain (cm/wk)	Moderate (some differences between Gps)
Huston 2018 [28] USA Some overlap of infants between this report and Huston 2014	Interrupted time series	379 infants Gp1: 1025 ± 164; 28.4 ± 1.9; 54 Gp2: 944 ± 199; 27.4 ± 2.0; 87 Gp3: 959 ± 174; 27.1 ± 2.0; 111 Gp4: 855 ± 209; 26.2 ± 2.2; 33 Gp5: 904 ± 200; 26.6 ± 2.4; 94	Gp1: PTF (MOM + PTF + BovF) (NR) Gp2: HMBF1 (MOM + DHM + BovF) (before implementation of the feeding protocol) (100%) Gp3: HMBF2 (MOM + DHM + BovF) (after implementation of the protocol) Gp4: EHM1 (MOM + HMDF) (before implementation of the feeding protocol) (100%) Gp5: EHM2 (MOM + HMDF) (after implementation of the feeding protocol)	C3: Gp2 + Gp4 vs. Gp1	Hospital stay	Growth (during hospital stay): change in wt, HC and length z-score	Moderate (some differences between Gps)
Jacobi-Polishook 2016 [37] Australia	Secondary analysis of RCT	611 infants median (range) Gp1: 1580 (720, 2280); 31.0 (25.0, 32.0); 46 Gp2: 1360 (530, 2620); 30.0 (24.0, 32.0); 141 Gp3: 1390 (420, 2400); 30.0 (23.0, 33.0); 141 Gp4: 1290 (500, 2090); 29.0 (23.0, 33.0); 142 Gp5: 1240 (320, 2480); 30.0 (24.0, 33.0); 141	Gp1: EPTF (0%) Gp2: Q1 (HM median (range) 49 (0.1, 85) mL/kg/d) Gp3: Q2 (HM 103 (85, 114) ml/kg/d) Gp4: Q3 (HM 124 (114, 134) ml/kg/d) Gp5: Q4 (HM 149 (134, 180) ml/kg/d)	C2: Gp2 + Gp3 + Gp4 + Gp5 vs. G1 C3: Gp4 + Gp5 vs. Gp2 + Gp3 BMI gain synthesised narratively	Hospital stay	Growth (from birth to DC): length gain (cm/wk), BMI gain	Moderate (some differences between Gps)
Kaempf 1998 [36] Germany	Non-randomised trial	19 infants Gp1: 1220 ± 310; 29 ± 1.1; 11 Gp2: 1220 ± 270; 30 ± 1.6; 8	Gp1: PTF (>80% PTF) Gp2: HM (fortified MOM) (>80% fortified MOM)	C3: Gp1 vs. Gp2 for wt gain (g/d); lower leg length synthesised narratively	SS: Gp1; age 8 ± 5 d, Gp2; age 10 ± 5 d Study duration: Gp1; 51 ± 12 d, Gp2; 48 ± 18 d	Growth (time frame for measures NR): wt gain (g/d), lower leg length (mm/d)	Moderate (some differences between Gps)
Lee 2020 [49] Singapore, Malaysia	Cohort	236 infants Gp 1: 855 ± 123; 27.1 ± 1.9; 40 Gp 2: 849 ±120; 27.6 ± 2.4; 34	Gp1 (Singapore): MOM 97% Gp2 (Malaysia): MOM 26%, Mix MOM/PTF 62%, EPTF 12%	C3: Gp1 vs. Gp2	Birth to 36 wk PMA	Growth (birth to 36 wk PMA): change in wt z-score	Low
Levene 2020 [50] United Kingdom	Cohort	193 infants Infants in ‘after’ cohort: 1117 ± 335; 28.1 ± 2.2; 209	Gp1: Exclusive HM (DHM until 34 wks PMA) (+package of key nutritional changes) Gp2: Any PTF (+package of key nutritional changes)	C3: Gp1 vs. Gp2	Hospital stay	Growth (birth to DC): wt gain (g/kg/d); change in wt z-score	Low
Li 2019 [38] United Kingdom	Secondary analysis of RCT	133 infants Gp1: 997 (780, 1178); 28.1 (26.5, 29.5); 56 Gp2: 1140 (885, 1398); 28.6 (26.8, 30.1); 39 Gp3: 1132 (905, 1334); 28.2 (26.9, 30.0); 38	(RCT: four PN intervention groups) Gp1: EHM; MOM (100%) Gp2: Predominantly HM; MOM + DHM + PTF (NR) Gp3: Predominantly PTF; MOM + DHM + PTF (NR)	C3: % FFM Gp1 + Gp2 vs. Gp 3; remainder of outcomes synthesised narratively	Nutritional intake: from birth until 34 wks PMA	Growth (birth to TCA): wt, length, and HC change in z-scores Body composition (MRI) at TCA: FM (g and %), FFM (g), FFM % (reported as median IQR and converted to mean, SD)	Low
Lok 2017 [22] Hong Kong	Cohort	175 VLBW infants Gp1: 1269.3 ± 180.6; NR; 31 Gp2: 1139.2 ± 205.9; NR; 144 Gp3: 1213 ± 204.3; NR; 55 Gp4: 1202 ± 189.5; NR; 25 Gp5: 1135 ± 221.5; NR; 47 Gp6: 1106 ± 191.4; NR; 47	Gp1: EPTF (0%) Gp2: Any HM (NR) Group by proportion of breast milk intake Gp3: <25% HM Gp4: 25–50% HM Gp5: 50–75% HM Gp6: >75% HM	C2: Gp2 vs. Gp1 C3: Gp5 + Gp6 vs. Gp3 + Gp4	The first 30 d of hospitalization	Growth (from birth to DC): change in wt and HC z-score	Low
Maas 2013 [51] Germany	Cohort	206 infants Gp1: 846 (705, 1160); 28.6 (25.5, 30.5); 37 Gp2: 925 (665, 1175); 27.6 (25.5, 29.7); 122	Gp1: <25% HM Gp2: >75% HM	C3: Gp 2 vs. Gp1	Hospital stay	Growth (from birth to day 28): wt and HC changes in z-score (reported as median, IQR and converted to mean, SD)	Moderate (some loss to FU)
Madore 2017 [52] USA	Cohort	81 infants Gp1: 936.6 ± 211.0; 27.0 ± 1.5; 29 Gp2: 913.8 ± 222.6; 27.3 ± 2.1; 25 Gp3: 890.5 ± 175.8; 27.1 ± 1.9; 27	Gp1: EHM; MOM (100%) Gp2: Predominantly PTF (>50% PTF) Gp3: DHM (>50% DHM)	C3: Gp1 + Gp3 vs. Gp2	The first month of life	Growth (from birth to day 30 and 60): wt gain (g/kg/d), HC gain (cm/wk), length gain (cm/wk)	Low
Manea 2016 [53] Romania	Cohort	34 infants Birth wt range; 850–1000 g Birth GA; 25–33 weeks Gp1: *n* = 16 Gp2: *n* = 18	Gp1: EPTF (0%) Gp2: EHM; MOM + BovF (after reached 100 mL/kg/day) (100%)	C1: synthesised narratively	Hospital stay	Growth (from birth to 5 wk of age): wt gain (g/d)	High (Gp characteristics and participant flow not described. SD and *p* values NR)
Mol 2019 [54] Poland	Cohort	53 infants BW; mean ± SD, GA; median (IQR) Gp1: 1240 ± 180; 29 (28–31.8); 23 Gp2: 1210 ± 161; 29 (28–32); 11 Gp3: 3320 ± 399; 39 (37–40); 19	Gp1: EPTF (0%) Gp2: Fortified HM (MOM + BovF) (100%) Gp3: full-term infants	C1: Gp2 vs. Gp1	Hospital stay	Body composition (BIS) at TCA: FM (g and %), FFM (g and %)	Moderate (milk amount not described)
Morlacchi 2018 [55] Italy	Cohort	32 infants Gp1: 1214.8 ± 246; 29.2 ± 1.6; 17 Gp2: 1293.0 ± 138; 30.3 ± 1; 15	Gp1: Fortified HM (MOM + BovF) (100%) Gp2: EPTF (0%)	C1: Gp1 vs. Gp2 Change in z-scores synthesised narratively	SS: at DC SE: at TCA Intervention start from birth to DC	Growth (from birth to DC): wt, length, and HC change in z-scores Body composition (ADP (PeaPod) at DC): FM (g and %), FFM (g and %)	Low
Nicholl 1999 [35] United Kingdom	RCT with non-randomised arm	52 infants Gp1: 1074 ± 216; 29 ± 2.1; 10 Gp2: 1002 ± 286; 28.1 ±2.4; 13 Gp3: 1087 ± 252; 28.7 ± 2.5; 29	Gp1: EHM (MOM + DHM) (100%) Gp2: EHM (MOM + DHM + BovF) (100%) Gp3: EPTF (0%) (not randomised)	C1: Gp2 vs. Gp3 Lower leg length synthesised narratively	SS: enteral feed ≥ 150 mL/kg/day SE: nasogastric feeds ceased	Growth (from SS to SE): wt gain (g/kg/d), lower leg length gain	Low
O’Connor 2001 [20] USA and United Kingdom	RCT with non-randomised reference group	470 infants Gp1: 1287 ± 272; 29.6 ± 1.9; 142 Gp2: 1305 ± 293; 29.8 ± 2.1; 138 Gp3: 1309 ± 286; 29.7 ± 2.0; 140 Gp4: 1275 ± 312; 29.7 ± 2.1; 43	Gp1: HM + PTF (NR) Gp2: HM + PTF + AA + DHA from fish/fungal oil Gp3: HM + PTF + AA + DHA from egg-derived triglyceride/fish oil G4: HM (>80% at term corrected age) (reference, not randomised)	C3: Gp4 vs. Gp1	SS: first enteral feed SE: 12 m CA	Growth (from SS to TCA): wt gain(g/kg/d), HC gain (cm/wk), length gain (cm/wk)	Low
Petrova 2020 [56] USA	Cohort	84 infants Gp 1: 1027 (95% CI 924, 1321); 27.7 (95% CI 26.8, 28.6); 37 Gp 2: 1285 (95% CI 1130, 1439); 29.8 (95% CI 28.6, 31.0); 16 Gp 3: 1272 (95% CI 1102, 1442); 29.1 (95% CI 28.2, 30.3); 31	Gp1: Predominantly HM (≥97% HM) Gp2: Partial HM (55–70% HM) Gp3: Predominantly PTF (≤9% HM)	C3: Gp1 + Gp2 vs. Gp3	SS: full enteral feeding had been achieved SE: 2 wks post full enteral feeds	Growth (from SS to SE): wt gain (g/kg/d) reported as median, IQR in figure format only; HC gain (cm/wk) reported as mean, 95% CI and converted to mean, SD	Moderate (some differences between Gps)
Pieltain 2001 [57] Belgium	Cohort	54 infants Gp1: 1298 ± 317; 31 ± 2; 20 Gp2: 1269 ± 261; 30 ± 2; 34	Gp1: Fortified HM; MOM + DHM + BovF (100%) Gp2: EPTF (0%)	C1: Gp1 vs. Gp2	SS: full enteral feeding had been achieved SE: at DC	Growth (from SS to SE): wt gain (g/kg/d), HC gain (cm/wk), length gain (cm/wk) Body composition (DEXA at SS and around DC): FFM (g), FM (g and %)	Low
Piemontese, 2018 [58] Italy	Cohort	73 infants Gp1: 1207 ± 208; 30 ± 2.4; 24 Gp2: 1269 ± 193; 30.3 ± 1.8; 49	Gp1: Fortified HM <50% intake; MOM + DHM + BovF + PTF (34.9% ± 12.5) Gp2: Fortified HM ≥50% intake; MOM + DHM + BovF + PTF (80.9% ± 15.5)	C3: Gp2 vs. Gp1	Hospital stay Targeted fortification commenced when intake ≥80 mL/kg	Growth (from birth to TCA): wt z-score SE, wt gain g/kg/day Body composition (ADP at TCA); FM (%), FFM (%)	Low
Schanler 1999 [9] USA	Secondary analysis of RCT	108 infants Gp1: 1069 ± 169; 27.9 ± 1.2; 62 Gp2: 1044 ± 185; 27.9 ± 1.1; 46	Gp1: Fortified HM; MOM + BovF (84 ± 20%, median 93%) Gp2: EPTF (0%)	C2: Gp1 vs. Gp2 Knee-heel length synthesised narratively	Hospital stay	Growth (from minimum wt to DC): wt gain (g/kg/d), HC gain (cm/wk), length gain (cm/wk)	Low
Simmer 1997 [59] Australia	Cohort	86 infants Gp1: 1486 ± 450; 30.8 ± 2.6, 43 Gp2: 1379 ± 347; 29.8 ± 2.5; 43	Gp1: Predominantly MOM; >50% (84 ± 15%) Gp2: Predominantly PTF; >50% PTF (16 ± 17%)	C3: Gp1 vs. Gp2	Hospital stay	Growth: wt gain (from week 2 to DC, g/kg/day), HC gain (during admission in the neonatal unit, cm/wk), length gain (during admission in the neonatal unit, cm/wk)	Low
Sisk 2008 [60] USA	Cohort	127 infants Gp1: 978 ± 149; 27.8 ± 2.1; 34 Gp2: 1000 ± 149; 27.4 ± 1.6; 93	Gp1: Lower HM (<50% of HM) Gp2: Higher HM (≥50% of HM)	C3: Gp 1 vs. Gp 2	Hospital stay	Growth (from regained BW to DC): wt gain (g/kg/day) (reported as median, IQR, and converted to mean, SD)	Low
Sisk 2017 [61] USA	Cohort	551 infants Gp1: 1017 ±291; 27.8 ± 2.4; 299 Gp2: 1026 ± 270; 28.0 ± 2.4; 139 Gp3: 1036 ± 21; 28.1 ± 2.6; 113	Gp1: MOM; ≥50% MOM (MOM 97%) Gp2: PDHM; ≥50% PDHM (PDHM 86%) Gp3: PTF; ≥50% PTF (PTF 91%)	C3: Gp1 + Gp2 vs. Gp3	SS: birth SE: NEC diagnosis or 34 wk PMA	Growth (from birth to DC): wt gain (g/kg/day), HC gain (cm/wk), length gain (cm/wk), change in z-scores (reported as median, IQR and converted to mean, SD)	Low
Soldateli 2020 [62] USA	Cohort (secondary analysis of data collected for QI initiative)	1429 infants Whole cohort: 1080 (861, 1285); 28 (27, 30)	Gp1: 0–25% HM Gp2: 26–50% HM Gp3: 51–75% HM Gp4: 76–99% Gp5: 100% HM Diet recorded on days of life 7, 14, 21, 28, 42, 56, 70, 84, and at discharge or transfer	C3: synthesised narratively	Birth to DC or transfer	Growth (from birth to DC or transfer): wt gain (g/kg/day), change in wt and length z-scores (only mean reported in text)	Moderate (unable to determine if Gps comparable)
Spielger 2016 [63] Germany	Cohort	1433 infants Gp1: 1080 (830, 1330); 28.7 (26.6, 30.1); 239 Gp2: 1100 (865, 1340); 29.0 (26.9, 30.0); 223 Gp3: 1050 (805, 1295); 28.4 (26.6, 30.0); 971	Gp1: EPTF; PTF (0%) Gp2: EHM; MOM + DHM (100%) Gp3: Mixed; MOM + DHM + PTF (NR)	C1: Gp1 vs. Gp2 C2: Gp2 + Gp3 vs. Gp1 C3: Gp2 vs. Gp1 + Gp2	Hospital stay	Growth (from birth to DC): change in wt z-score, (reported as median, IQR, and converted to mean, SD)	Low
Verd 2015 [42] Spain	Interrupted time series	201 infants Gp1: 800 (410, 995); GA (d) 185 (161, 236); 148 Gp2: 830 (440, 998); 190 (166, 239); 53	Gp1: EHM; MOM + DHM (100%) Gp2: Any PTF; MOM + DHM + PTF (NR)	C3: Gp1 vs. Gp2	Hospital stay	Growth (from birth to DC): change in wt, length, and HC z-scores reported as median, IQR, and converted to mean, SD	Low
Warner 1998 [64] United Kingdom	Cohort	59 infants, median(range) Gp1: 1178 (685, 1510); 29.6 (25, 33); 38 Gp2: 1120 (840, 1580); 29.1 (25, 34); 21	Gp1: HM; MOM + BovF + (term infant formula if MOM was insufficient) (NR) Gp2: EPTF (0%)	C2: Gp1 vs. Gp2	SS: at birth SE: wt of 1800 g was reached	Growth: wt gain (from start of full feeds to 1800 g, g/kg/d), HC gain (from SS to SE, cm/wk), length gain (from SS to SE, cm/wk)	Low
Wauben 1998 [21,65] Canada	RCT with non-randomised reference group	37 infants Gp1: 1400 ± 200; 29.9 ± 1.9; 12 Gp2: 1300 ± 200; 30.1 ± 1.5; 13 Gp3: 1200 ± 200; 29.7 ± 1.7, 12	Gp1: MOM + multinutrient fortifier (100%) Gp2: MOM + calcium and phosphorus alone (100%) Gp3: PTF (0%) (comparison group, not randomised)	C1: Gp1 vs. Gp3 Body composition C3: Gp1 vs. Gp3 (Gp2 not fortified therefore excluded)	SS: full oral feeds ≥5 d SE: Discharge home or >38 wk PMA (whichever came first)	Growth (from SS to DC): wt gain (g/kg/d), HC gain (cm/wk), length gain (cm/wk) [21] Body composition (DXA) at TCA: FM (%), FFM (%) [65]	Low

Data presented as mean ± SD or median (IQR) unless otherwise stated. Abbreviations: AA, arachidonic acid; ADP, air displacement plethysmography; BIS, bioelectrical impedance spectroscopy; BMI, Body mass index; BovF, Bovine fortifier; BW, birth weight; CA, corrected age; d, days; DC, discharge; DEXA, dual energy x-ray absorptiometry; DHA, docosahexaenoic acid; DHM, donor human milk; EHM, exclusive human milk; EPTF, exclusive preterm formula; FFM, fat free mass; FM, fat mass; FU, follow-up; GA, gestational age; Gp, group; HC, head circumference; HM, human milk; HMDF, human milk derived fortifier; IQR, inter quartile range; LCPUFA, long chain polyunsaturated fatty acids; m, month; MOM, mother’s own milk; NEC, necrotising enterocolitis; NR, not reported; PDHM, pasteurised donor human milk; PMA, post menstrual age; PN, parenteral nutrition; PTF, preterm formula; Q, quartile; QI, quality improvement; RCT, randomised controlled trial; SD, standard deviation; SE, study end; SS, study start; TCA, term corrected age; VLBW, very low birth weight; wk, weeks; wt, weight.

**Table 2:** Summary of findings.

Outcome	EPTF vs. EHM Anticipated Absolute Effects MD (95% CI); *N* Participants; *N* Studies GRADE Certainty of Evidence Interpretation	EPTF vs. Any HM Anticipated Absolute Effects MD (95% CI); *N* Participants; *N* Studies GRADE Certainty of Evidence Interpretation	Low- vs. High-Dose HM Anticipated Absolute Effects MD (95% CI); *N* Participants; *N* Studies GRADE Certainty of Evidence Interpretation
**WEIGHT GAIN, G/DAY**	Randomised controlled trial MD 2 (−1.54 to 5.54); *n* = 53; 1 study Certainty: not graded; ROB moderate Interpretation: Inconclusive	No studies identified	Observational studies MD −0.83 (−1.65 to 0); *n* = 1606; 2 studies Certainty: low Interpretation: Possible effect of a small decrease in weight gain (g/day) with lower-dose HM versus higher-dose HM
**WEIGHT GAIN, G/KG/DAY**	Observational studies MD 2.03 (−0.31 to 4.38); *n* = 364; 4 studies Certainty: very low Interpretation: Inconclusive	Observational studies MD 1.97 (0.21 to 3.72); *n* = 795; 5 studies Certainty: very low Interpretation: Inconclusive	Randomised controlled trial MD 2.41 (1.09 to 3.72); *n* = 373; 2 studies Certainty: low Observational studies MD 0.56 (0.09 to 1.03); *n* = 3162; 13 studies; Certainty: very low Interpretation: Possible effect of a small increase in weight gain (g/kg/day) with lower-dose HM versus high-dose HM
**CHANGE IN WEIGHT Z-SCORE**	Observational studies MD 0.26 (0.03 to 0.48); *n* = 49; 2 studies Certainty: low Interpretation: Possible effect of a small increase in weight z-score with EPTF vs. EHM	Observational studies MD 0.21 (−0.15 to 0.56); *n* = 1532; 3 studies Certainty: very low Interpretation: Inconclusive	Randomised controlled trial MD 0 (−0.29 to 0.29); *n* = 326; 1 study Certainty: not graded; ROB low Observational studies MD 0.19 (0.6 to 0.33); *n* = 4059; 12 studies Certainty: very low Interpretation: Inconclusive
**HC GAIN, CM/WK**	Randomised controlled trial MD 0.1 (−0.02 to 0.22); *n* = 53; 1 study Certainty: not graded; ROB moderate Observational studies MD 0.09 (−0.10 to 0.29); *n* = 78; 2 studies Certainty: very low Interpretation: Inconclusive	Observational studies MD 0.06 (0.1 to 0.11); *n* = 495; 4 studies Certainty: low Interpretation: Possible effect of small increase in HC gain in infants fed EPTF versus any human milk	Randomised controlled trial MD 0 (−0.06 to 0.06); *n* = 373; 2 studies Certainty: moderate Observational Studies MD 0.04 (0.02 to 0.07); *n* = 4080; 10 studies Certainty: very low Interpretation: Possibly no effect of HM dose on HC gain (cm/wk)
**CHANGE IN HC Z-SCORE**	Observational study MD 0.1 (−0.42 to 0.62); *n* = 32, 1 study Certainty: not graded; ROB low Interpretation: Inconclusive	Observational studies MD 0.43 (0.18 to 0.69); *n* = 322; 2 studies Certainty: low Interpretation: Possible effect of small increase in HC z-score with EPTF versus any HM.	Randomised controlled trial MD 0.2 (−0.08 to 0.48); *n* = 326; 1 study Certainty: not graded; ROB low Observational studies MD 0.09 (−0.19 to 0.38); *n* = 2627; 8 studies Certainty: very low Interpretation: Inconclusive
**LENGTH GAIN, CM/WK**	Randomised controlled trial MD 0.28 (0.14 to 0.42); *n* = 53; 1 study Certainty: not graded; ROB moderate Observational studies MD 0.06 (−0.07 to 0.19); *n* = 78; 2 studies Certainty: very low Interpretation: Inconclusive	Observational studies MD 0.09 (−0.05 to 0.22); *n* = 778; 3 studies Certainty: very low Interpretation: Inconclusive	Randomised controlled trial MD −0.04 (−0.28 to 0.21); *n* = 373; 2 studies Certainty: low Observational studies MD 0.05 (0.02 to 0.08); *n* = 2423; 8 studies Certainty: low Interpretation: Possibly no effect of dose of human milk on length gain (cm/wk)
**CHANGE IN LENGTH Z-SCORE**	Observational study MD 0.0 (−0.63 to 0.63); *n* = 32, 1 study Certainty: not graded; ROB low Interpretation: Inconclusive	No studies detected	Randomised controlled trial MD 0.1 (−0.26 to 0.46); *n* = 326; 1 study Certainty: not graded; ROB low Observational study MD 0.09 (−0.07 to 0.25); *n* = 1131, 3 studies Certainty: very low Interpretation: Inconclusive
**FAT FREE MASS %**	Observational studies MD −1.46 (−4.35 to 1.43); *n* = 87; 3 studies Certainty: very low Interpretation: Inconclusive	No studies identified	Observational studies MD −5.1 (−12.45 to 2.25); *n* = 73; 1 study Certainty: not graded; ROB low Interpretation: Inconclusive
**FAT FREE MASS G**	Observational studies MD 130.18 (53.86 to 206.5); *n* = 134; 4 studies Certainty: very low Interpretation: Inconclusive	No studies identified	No studies identified
**FAT MASS %**	Observational studies MD 1.82 (−0.59 to 4.23); *n* = 141; 4 studies Certainty: very low Interpretation: Inconclusive	No studies identified	Observational studies MD −0.48 (−1.7 to 0.73); *n* = 133; 1 study Certainty: not graded; ROB low Interpretation: Inconclusive
**FAT MASS G**	Observational studies MD 60.94 (−5.42 to 127.31); *n* = 134; 4 studies Certainty: very low Interpretation: Inconclusive	No studies identified	No studied identified

Abbreviations: CI, confidence interval; EHM, exclusive human milk; EPTF, exclusive preterm formula; HC, head circumference; HM, human milk; MD, mean difference; RCT, randomised controlled trial; ROB, risk of bias; Interpretation: Clear effect/clear evidence of no effect: The certainty of evidence is moderate or above with a clinically important result from RCTs, ideally aligning with results from observational studies or moderate certainty evidence from observational studies; and with reasonable numbers of events and/or participants. Probably an effect/probably no effect: There is moderate certainty from either RCTs or observational studies and point estimates may be different between the 2 study types with overlapping CIs but can be explained (e.g., through heterogeneity). There are large numbers of participants and studies. Possible effect/possibly no effect: There is low/ moderate certainty with CIs which may suggest a difference although not reaching conventional statistical significance; or with a confidence interval that indicates a trivial difference only. Inconclusive: The certainty of evidence is very low to low, CIs are wide, and number of participants and studies is low.

## Supplementary Materials

The following are available online at https://www.mdpi.com/article/10.3390/nu13062089/s1, Figure S1: Prisma diagram—selection of studies, Table S1: Results of studies synthesised narratively, Table S2: Summary of findings: Preterm formula vs. Human milk—Weight gain (g/d), Table S3: Summary of findings: Preterm formula vs. Human milk—Weight gain (g/kg/d), Table S4: Summary of findings: Preterm formula vs. Human milk—Change in weight z-score, Table S5: Summary of findings: Preterm formula vs. Human milk title—Head circumference gain, Table S6: Summary of findings: Preterm formula vs. Human milk—Change in head circumference z-score, Table S7: Summary of findings: Preterm formula vs. Human milk—Length gain (cm/week), Table S8: Summary of findings: Preterm formula vs. Human milk—Change in length z-score, Table S9: Summary of findings: Preterm formula vs. Human milk—% Fat-free mass, Table S10: Summary of findings: Preterm formula vs. Human milk—Fat-free mass (g), Table S11: Summary of findings: Preterm formula vs. Human milk—% fat mass, Table S12: Summary of findings: Preterm formula vs. Human milk—Fat mass (g).

## References

[1] Edmond K., Bahl R. (2006). Optimal Feeding of Low-Birth-Weight Infants.

[2] Agostoni C., Buonocore G., Carnielli V.P., de Curtis M., Darmaun D., Decsi T., Domellof M., Embleton N.D., Fusch C., Genzel-Boroviczeny O. (2010). Enteral nutrient supply for preterm infants: Commentary from the european society of paediatric gastroenterology, hepatology and nutrition committee on nutrition. J. Pediatr. Gastroenterol. Nutr..

[3] Miller J., Tonkin E., Damarell R., McPhee A., Suganuma M., Suganuma H., Middleton P., Makrides M., Collins C. (2018). A systematic review and meta-analysis of human milk feeding and morbidity in very low birth weight infants. Nutrients.

[4] AAP Committee on Nutrition, AAP Section on Breastfeeding, AAP Committee on Fetus and Newborn (2017). Donor human milk for the high-risk infant: Preparation, safety, and usage options in the United States. Pediatrics.

[5] Brown J.V., Lin L., Embleton N.D., Harding J.E., McGuire W. (2020). Multi-nutrient fortification of human milk for preterm infants. Cochrane Database Syst. Rev..

[6] Arslanoglu S., Boquien C.-Y., King C., Lamireau D., Tonetto P., Barnett D., Bertino E., Gaya A., Gebauer C., Grovslien A. (2019). Fortification of human milk for preterm infants: Update and recommendations of the european milk bank association (emba) working group on human milk fortification. Front. Pediatr..

[7] Colaizy T.T., Carlson S., Saftlas A.F., Morriss F.H. (2012). Growth in vlbw infants fed predominantly fortified maternal and donor human milk diets: A retrospective cohort study. BMC Pediatr..

[8] Ginovart G., Gich I., Gutierrez A., Verd S. (2017). A fortified donor milk policy is associated with improved in-hospital head growth and weight gain in very low-birth-weight infants. Adv. Neonatal Care.

[9] Schanler R.J., Shulman R.J., Lau C. (1999). Feeding strategies for premature infants: Beneficial outcomes of feeding fortified human milk versus preterm formula. Pediatrics.

[10] Brown J.V.E., Walsh V., McGuire W. (2019). Formula versus maternal breast milk for feeding preterm or low birth weight infants. Cochrane Database Syst. Rev..

[11] Quigley M., Embleton N.D., McGuire W. (2019). Formula versus donor breast milk for feeding preterm or low birth weight infants. Cochrane Database Syst. Rev..

[12] Huang P., Zhou J., Yin Y., Jing W., Luo B., Wang J. (2016). Effects of breast-feeding compared with formula-feeding on preterm infant body composition: A systematic review and meta-analysis. Br. J. Nutr..

[13] Leppanen M., Lapinleimu H., Lind A., Matomaki J., Lehtonen L., Haataja L., Rautava P., Group P.S. (2014). Antenatal and postnatal growth and 5-year cognitive outcome in very preterm infants. Pediatrics.

[14] Moher D., Liberati A., Tetzlaff J., Altman D.G., Group P. (2009). Preferred reporting items for systematic reviews and meta-analyses: The prisma statement. BMJ.

[15] Costa-Orvay J.A., Figueras-Aloy J., Romera G., Closa-Monasterolo R., Carbonell-Estrany X. (2011). The effects of varying protein and energy intakes on the growth and body composition of very low birth weight infants. Nutr. J..

[16] Belfort M.B., Rifas-Shiman S.L., Sullivan T., Collins C.T., McPhee A.J., Ryan P., Kleinman K.P., Gillman M.W., Gibson R.A., Makrides M. (2011). Infant growth before and after term: Effects on neurodevelopment in preterm infants. Pediatrics.

[17] Cormack B.E., Embleton N.D., van Goudoever J.B., Hay W.W., Bloomfield F.H. (2016). Comparing apples with apples: It is time for standardized reporting of neonatal nutrition and growth studies. Pediatr. Res..

[18] Covidence Systematic Review Software.

[19] Fewtrell M.S., Morley R., Abbott R.A., Singhal A., Isaacs E.B., Stephenson T., MacFadyen U., Lucas A. (2002). Double-blind, randomized trial of long-chain polyunsaturated fatty acid supplementation in formula fed to preterm infants. Pediatrics.

[20] O’Connor D.L., Hall R.T., Adamkin D.H., Auestad N., Castillo M., Connor W.E., Connor S.L., Fitzgerald K.M., Groh-Wargo S., Hartmann E.E. (2001). Growth and development in preterm infants fed long-chain polyunsaturated fatty acids: A prospective, randomized controlled trial. Pediatrics.

[21] Wauben I.P., Atkinson S.A., Grad T.L., Shah J.K., Paes B. (1998). Moderate nutrient supplementation of mother’s milk for preterm infants supports adequate bone mass and short-term growth: A randomized, controlled trial. Am. J. Clin. Nutr..

[22] Lok K.Y.W., Chau P.H., Fan H.S.L., Chan K.M., Chan B.H., Fung G.P.C., Tarrant M. (2017). Increase in weight in low birth weight and very low birth weight infants fed fortified breast milk versus formula milk: A retrospective cohort study. Nutrients.

[23] Higgins J.P.T., Thomas J., Chandler J., Cumpston M., Li T., Page M.J., Welch V.A. Cochrane Handbook for Systematic Reviews of Interventions.

[24] Academy of Nutrition and Dietetics (2016). Evidence Analysis Manual: Steps in the Academy Evidence Analysis Process.

[25] (2020). Review Manager (RevMan) [Computer Program].

[26] Luo D., Wan X., Liu J., Tong T. (2016). Optimally estimating the sample mean from the sample size, median, mid-range, and/or mid-quartile range. Stat. Methods Med. Res..

[27] Wan X., Wang W., Liu J., Tong T. (2014). Estimating the sample mean and standard deviation from the sample size, median, range and/or interquartile range. BMC Med. Res. Methodol..

[28] Huston R.K., Markell A.M., McCulley E.A., Gardiner S.K., Sweeney S.L. (2018). Improving growth for infants ≤250 grams receiving an exclusive human milk diet. Nutr. Clin. Pract..

[29] Huston R.K., Markell A.M., McCulley E.A., Pathak M., Rogers S.P., Sweeney S.L., Dolphin N.G., Gardiner S.K. (2014). Decreasing necrotizing enterocolitis and gastrointestinal bleeding in the neonatal intensive care unit: The role of donor human milk and exclusive human milk diets in infants ≤1500 g birth weight. Infant Child. Adolesc. Nutr..

[30] (2015). Gradepro Gdt: Gradepro Guideline Development Tool [Software].

[31] Cristofalo E.A., Schanler R.J., Blanco C.L., Sullivan S., Trawoeger R., Kiechl-Kohlendorfer U., Dudell G., Rechtman D.J., Lee M.L., Lucas A. (2013). Randomized trial of exclusive human milk versus preterm formula diets in extremely premature infants. J. Pediatr..

[32] O’Connor D.L., Gibbins S., Kiss A., Bando N., Brennan-Donnan J., Ng E., Campbell D.M., Vaz S., Fusch C., Asztalos E. (2016). Effect of supplemental donor human milk compared with preterm formula on neurodevelopment of very low-birth-weight infants at 18 months: A randomized clinical trial. JAMA.

[33] Schanler R.J., Lau C., Hurst N.M. (2005). Randomized trial of donor human milk versus preterm formula as substitutes for mothers’ own milk in the feeding of extremely premature infants. Pediatrics.

[34] Sullivan S., Schanler R.J., Kim J.H., Patel A.L., Trawöger R., Kiechl-Kohlendorfer U., Chan G.M., Blanco C.L., Abrams S., Cotten C.M. (2010). An exclusively human milk-based diet is associated with a lower rate of necrotizing enterocolitis than a diet of human milk and bovine milk-based products. J. Pediatr..

[35] Nicholl R.M., Gamsu H.R. (1999). Changes in growth and metabolism in very low birthweight infants fed with fortified breast milk. Acta Paediatr..

[36] Kaempf D.E., Pfluger M.S., Thiele A.M., Hermanussen M., Linderkamp O. (1998). Influence of nutrition on growth in premature infants: Assessment by knemometry. Ann. Hum. Biol..

[37] Jacobi-Polishook T., Collins C.T., Sullivan T.R., Simmer K., Gillman M.W., Gibson R.A., Makrides M., Belfort M.B. (2016). Human milk intake in preterm infants and neurodevelopment at 18 months corrected age. Pediatr. Res..

[38] Li Y., Liu X., Modi N., Uthaya S. (2019). Impact of breast milk intake on body composition at term in very preterm babies: Secondary analysis of the nutritional evaluation and optimisation in neonates randomised controlled trial. Arch. Dis. Child. Fetal. Neonatal Ed..

[39] Assad M., Elliott M.J., Abraham J.H. (2016). Decreased cost and improved feeding tolerance in vlbw infants fed an exclusive human milk diet. J. Perinatol..

[40] Colacci M., Murthy K., DeRegnier R.O., Khan J.Y., Robinson D.T. (2017). Growth and development in extremely low birth weight infants after the introduction of exclusive human milk feedings. Am. J. Perinatol..

[41] Hoban R., Schoeny M.E., Esquerra-Zwiers A., Kaenkumchorn T.K., Casini G., Tobin G., Siegel A.H., Patra K., Hamilton M., Wicks J. (2019). Impact of donor milk on short- and long-term growth of very low birth weight infants. Nutrients.

[42] Verd S., Porta R., Botet F., Gutierrez A., Ginovart G., Barbero A.H., Ciurana A., Plata (2015). Hospital outcomes of extremely low birth weight infants after introduction of donor milk to supplement mother’s milk. Breastfeed. Med..

[43] Brownell E.A., Matson A.P., Smith K.C., Moore J.E., Esposito P.A., Lussier M.M., Lerer T.J., Hagadorn J.I. (2018). Dose-response relationship between donor human milk, mother’s own milk, preterm formula, and neonatal growth outcomes. J. Pediatr. Gastroenterol. Nutr..

[44] Cañizo Vázquez D., Salas García S., Izquierdo Renau M., Iglesias-Platas I. (2019). Availability of donor milk for very preterm infants decreased the risk of necrotizing enterocolitis without adversely impacting growth or rates of breastfeeding. Nutrients.

[45] Carlson S.J., Ziegler E.E. (1998). Nutrient intakes and growth of very low birth weight infants. J. Perinatol..

[46] Castellano Yáñez C., Castillo Barrio B., Muñoz Labián M.D.C., Ortiz Movilla R., García Lara N.R., Royuela Vicente A., Marín Gabriel M.A. (2019). Providing very preterm infants with donor human milk led to faster breastfeeding rates but worse biometric gains. Acta Paediatr..

[47] Chowning R., Radmacher P., Lewis S., Serke L., Pettit N., Adamkin D.H. (2016). A retrospective analysis of the effect of human milk on prevention of necrotizing enterocolitis and postnatal growth. J. Perinatol..

[48] Hair A.B., Peluso A.M., Hawthorne K.M., Perez J., Smith D.P., Khan J.Y., O’Donnell A., Powers R.J., Lee M.L., Abrams S.A. (2016). Beyond necrotizing enterocolitis prevention: Improving outcomes with an exclusive human milk–based diet. Breastfeed. Med..

[49] Lee L.Y., Lee J., Niduvaje K., Seah S.S., Atmawidjaja R.W., Cheah F.C. (2020). Nutritional therapies in the neonatal intensive care unit and post-natal growth outcomes of preterm very low birthweight asian infants. J. Paediatr. Child. Health.

[50] Levene I., McCormick K. (2020). Improved growth of extremely and very preterm babies: Evaluation of a quality-of-care initiative. J. Paediatr. Child. Health.

[51] Maas C., Wiechers C., Bernhard W., Poets C.F., Franz A.R. (2013). Early feeding of fortified breast milk and in-hospital-growth in very premature infants: A retrospective cohort analysis. BMC Pediatr..

[52] Madore L.S., Bora S., Erdei C., Jumani T., Dengos A.R., Sen S. (2017). Effects of donor breastmilk feeding on growth and early neurodevelopmental outcomes in preterm infants: An observational study. Clin. Ther..

[53] Manea A., Boia M., Iacob D., Dima M., Iacob R.E. (2016). Benefits of early enteral nutrition in extremely low birth weight infants. Singapore Med. J..

[54] Mol N., Zasada M., Kwinta P. (2019). Does type of feeding affect body composition in very low birth weight infants?—A prospective cohort study. Pediatr. Neonatol..

[55] Morlacchi L., Roggero P., Gianni M.L., Bracco B., Porri D., Battiato E., Menis C., Liotto N., Mallardi D., Mosca F. (2018). Protein use and weight-gain quality in very-low-birth-weight preterm infants fed human milk or formula. Am. J. Clin. Nutr..

[56] Petrova A., Eccles S., Mehta R. (2020). Role of the proportional intake of fortified mother’s own milk in the weight gain pattern of their very-preterm-born infants. Nutrients.

[57] Pieltain C., de Curtis M., Gerard P., Rigo J. (2001). Weight gain composition in preterm infants with dual energy x-ray absorptiometry. Pediatr. Res..

[58] Piemontese P., Liotto N., Mallardi D., Roggero P., Puricelli V., Gianni M.L., Morniroli D., Tabasso C., Perrone M., Menis C. (2018). The effect of human milk on modulating the quality of growth in preterm infants. Front. Pediatr..

[59] Simmer K., Metcalf R., Daniels L. (1997). The use of breastmilk in a neonatal unit and its relationship to protein and energy intake and growth. J. Paediatr. Child. Health.

[60] Sisk P.M., Lovelady C.A., Gruber K.J., Dillard R.G., O’Shea T.M. (2008). Human milk consumption and full enteral feeding among infants who weigh ≤1250 grams. Pediatrics.

[61] Sisk P.M., Lambeth T.M., Rojas M.A., Lightbourne T., Barahona M., Anthony E., Auringer S.T. (2017). Necrotizing enterocolitis and growth in preterm infants fed predominantly maternal milk, pasteurized donor milk, or preterm formula: A retrospective study. Am. J. Perinatol..

[62] Soldateli B., Parker M., Melvin P., Gupta M., Belfort M. (2020). Human milk feeding and physical growth in very low-birth-weight infants: A multicenter study. J. Perinatol..

[63] Spiegler J., Preuss M., Gebauer C., Bendiks M., Herting E., Gopel W., German Neonatal Network (2016). Does breastmilk influence the development of bronchopulmonary dysplasia?. J. Pediatr..

[64] Warner J.T., Linton H.R., Dunstan F.D., Cartlidge P.H. (1998). Growth and metabolic responses in preterm infants fed fortified human milk or a preterm formula. Int. J. Clin. Pract..

[65] Wauben I.P., Atkinson S.A., Shah J.K., Paes B. (1998). Growth and body composition of preterm infants: Influence of nutrient fortification of mother’s milk in hospital and breastfeeding post-hospital discharge. Acta Paediatr..

[66] Hamatschek C., Yousuf E.I., Mollers L.S., So H.Y., Morrison K.M., Fusch C., Rochow N. (2020). Fat and fat-free mass of preterm and term infants from birth to six months: A review of current evidence. Nutrients.

[67] Franz A.R., Pohlandt F., Bode H., Mihatsch W.A., Sander S., Kron M., Steinmacher J. (2009). Intrauterine, early neonatal, and postdischarge growth and neurodevelopmental outcome at 5.4 years in extremely preterm infants after intensive neonatal nutritional support. Pediatrics.

[68] Roze J.C., Darmaun D., Boquien C.Y., Flamant C., Picaud J.C., Savagner C., Claris O., Lapillonne A., Mitanchez D., Branger B. (2012). The apparent breastfeeding paradox in very preterm infants: Relationship between breast feeding, early weight gain and neurodevelopment based on results from two cohorts, epipage and lift. BMJ Open.

[69] Gao C., Miller J., Collins C.T., Rumbold A.R. (2020). Comparison of different protein concentrations of human milk fortifier for promoting growth and neurological development in preterm infants. Cochrane Database Syst. Rev..

[70] Premkumar M.H., Pammi M., Suresh G. (2019). Human milk-derived fortifier versus bovine milk-derived fortifier for prevention of mortality and morbidity in preterm neonates. Cochrane Database Syst. Rev..

[71] Grace E., Hilditch C., Gomersall J., Collins C.T., Rumbold A., Keir A.K. (2021). Safety and efficacy of human milk-based fortifier in enterally fed preterm and/or low birthweight infants: A systematic review and meta-analysis. Arch. Dis. Child. Fetal Neonatal Ed..

[72] John A., Sun R., Maillart L., Schaefer A., Spence E.H., Perrin M.T. (2019). Macronutrient variability in human milk from donors to a milk bank: Implications for feeding preterm infants. PLoS ONE.

[73] Paulaviciene I.J., Liubsys A., Eidukaite A., Molyte A., Tamuliene L., Usonis V. (2020). The effect of prolonged freezing and holder pasteurization on the macronutrient and bioactive protein compositions of human milk. Breastfeed. Med..

