# Supplementary material for: A Systematic Review and Meta-Analysis of Human Milk Feeding and Short-Term Growth in Preterm and Very Low Birth Weight Infants
Source: Nutrients. 2021 Jun 18;13(6):2089. doi: 10.3390/nu13062089 (PMC8234723; doi:10.3390/nu13062089)
Supplement: Supplementary file 1 [file nutrients-13-02089-s001.zip › nutrients-1235030-supplementary.pdf]

Supplementary Materials

Table of contents

Tables ... 2

Table S1. Results of studies synthesised narratively ... 2

Table S2. Summary of findings: Preterm formula vs Human milk – Weight gain (g/d) ... 6

Table S3. Summary of findings: Preterm formula vs Human milk – Weight gain (g/kg/d) ... 7

Table S4. Summary of findings: Preterm formula vs Human milk – change in weight z-score ... 8

Table S5. Summary of findings: Preterm formula vs Human milk - head circumference gain (cm/wk) ... 9

Table S6. Summary of findings: Preterm formula vs Human milk – change in head circumference z-score ... 10

Table S7. Summary of findings: Preterm formula vs Human milk – length gain (cm/week) ... 11

Table S8. Summary of findings: Preterm formula vs Human milk - change in length z-score ... 12

Table S9. Summary of findings: Preterm formula vs Human milk - % fat-free mass ... 13

Table S10. Summary of findings: Preterm formula vs Human milk - fat-free mass (g) ... 14

Table S11. Summary of findings: Preterm formula vs Human milk - % fat mass ... 15

Table S12. Summary of findings: Preterm formula vs Human milk - fat mass (g) ... 16

Figures ... 17

Figure S1. Prisma diagram – selection of studies ... 17

References ... 18

Tables

Table S1. Results of studies synthesised narratively

<table>
<tr><th>Study</th><th>Variable synthesised narratively</th><th>Reason for non-inclusion in meta-analyses</th><th>Comparison for this review</th><th>Results</th></tr>
<tr><td>Brownell 2018 [1]</td><td>Wt, length and HC gain, change in z-score</td><td>Multivariable linear regression models used for associations between feeding and growth. Feeding groups were not mutually exclusive</td><td>C3</td><td>
Associations between diet composition and growth velocity, using MOM as reference and 10% increases in PTF intake
<table>
<tr><th></th><th>Weight gain, g/kg/day
n=313
β (95% CI)</th><th>HC growth, cm/wk
n=307
β (95% CI)</th><th>Length gain, cm/wk
n=311
β (95% CI)</th></tr>
<tr><td>Adjusted mean for MOM</td><td>17.08 (12.7, 21.45)</td><td>1.17 (0.75, 1.58)</td><td>3.18 (2.25, 4.11)</td></tr>
<tr><td>Proportion of diet as DHM</td><td>-0.17 (-0.28, -0.05)
P=0.01</td><td>-0.01 (-0.02, -0.001)
P=0.03</td><td>-0.001 (-0.3, 0.03)
P=0.95</td></tr>
<tr><td>Proportion of diet as PTF</td><td>0.09 (-0.04, 0.21)
P=0.18</td><td>0.01 (-0.01, 0.020)
P=0.23</td><td>-0.01 (-0.05, 0.02)
P=0.44</td></tr>
</table>
Associations between diet composition and growth z-scores, using MOM as reference and 10% increases in PTF intake
<table>
<tr><th></th><th>Weight z-score change
n=314
β (95% CI) p</th><th>HC z-score change
n=312
β (95% CI) p</th><th>Length z-score change
n=298
β (95% CI) p</th></tr>
<tr><td>Adjusted mean change in z-score for MOM</td><td>-0.93 (-1.15, -0.72)</td><td>-0.31 (-0.51, -0.10)</td><td>-0.12 (-0.62, 0.40)</td></tr>
<tr><td>Proportion of diet as DHM</td><td>-0.04 (-0.06, -0.02)
P <0.001</td><td>-0.06 (-0.10, _0.02)
P= 0.002</td><td>-0.05 (-0.13, 0.03)
P= 0.22</td></tr>
<tr><td>Proportion of diet as PTF</td><td>0.03 (0.01, 0.05)
P= 0.01</td><td>0.002 (-0.04, 0.04)
P= 0.92</td><td>-0.09 (-0.17, -0.01)
P=0.04</td></tr>
</table>
</td></tr>
<tr><td>Castellano Yanez 2019 [2]</td><td>Wt, length, HC, change in z-score</td><td>Data reported as mean z-score difference over hospital stay between groups</td><td>C3</td><td>
Mean difference in z-score between higher dose HM (n=78) and lower dose HM (n=52) over hospital stay
<table>
<tr><th></th><th>MD (95% CI)</th><th>P</th></tr>
<tr><td>Weight z-score</td><td>-0.104 (-0.175 to -0.032)</td><td>0.004</td></tr>
<tr><td>Length z-score</td><td>-0.312 (-0.469 to -0.154)</td><td><0.001</td></tr>
<tr><td>HC z-score</td><td>-0.166 (-0.301 to -0.30)</td><td><0.017</td></tr>
</table>
</td></tr>
</table>

<table>
<thead>
<tr><th>Study</th><th>Variable synthesised narratively</th><th>Reason for non-inclusion in meta-analyses</th><th>Comparison for this review</th><th>Results</th></tr>
</thead>
<tbody>
<tr>
<td>Carlson 1998 [3]</td>
<td>Wt gain</td>
<td>Growth reported in time periods 0 -14 d, 15 – 35 d, 36 – 56 d, 57 d to TCA and not comparable with other data</td>
<td>C1, C2 & C3</td>
<td>Wt gain (mean ± SD) according to feeding type
<table>
<tr><th>Feeding type</th><th>N</th><th>Weight gain, g/kg/day</th></tr>
<tr><td>Age 0 – 14 days</td><td></td><td></td></tr>
<tr><td>EHM</td><td>23</td><td>1.7 ± 4.9</td></tr>
<tr><td>Mixed feeds</td><td>14</td><td>-0.1 ± 4.8</td></tr>
<tr><td>EPTF</td><td>14</td><td>2.4 ± 7.1</td></tr>
<tr><td>Age 15 – 35 days[1,2]</td><td></td><td></td></tr>
<tr><td>EHM</td><td>23</td><td>11.5 ± 4.0</td></tr>
<tr><td>Mixed feeds</td><td>14</td><td>13.2 ± 4.4</td></tr>
<tr><td>EPTF</td><td>14</td><td>15.9 ± 3.3</td></tr>
<tr><td>Age 36-56 days</td><td></td><td></td></tr>
<tr><td>EHM</td><td>21</td><td>12.2 ± 3.6</td></tr>
<tr><td>Mixed feeds</td><td>13</td><td>16.0 ± 4.1</td></tr>
<tr><td>EPTF</td><td>11</td><td>14.0 ± 3.2</td></tr>
<tr><td>Age 57 days to term[1,3]</td><td></td><td></td></tr>
<tr><td>EHM</td><td>15</td><td>9.9 ± 3.9</td></tr>
<tr><td>Mixed feeds</td><td>9</td><td>11.9 ± 2.8</td></tr>
<tr><td>EPTF</td><td>10</td><td>13.7 ± 4.3</td></tr>
</table>
[1] EPTF vs EHM P<0.05
[2] EPTF vs any HM MD 3.76 g/kg/d, 95% CI 1.57 to 5.95 (not reported in paper – calculated using Revman 4.1)
[3] EPTF vs any HM MD 3.05, 95% CI 0.02 to 6.08 (not reported in paper – calculated using Revman 4.1)
</td>
</tr>
<tr>
<td>Hoban 2019 [4]</td>
<td>Wt length and HC z-scores</td>
<td>Data reported as deviation from the reference growth trend for each week (NICU LOS, GA), female sex, and for each 10% increase in proportion of formula.</td>
<td>C3</td>
<td>
<table>
<tr><th></th><th>Weight (change in z-score) n=321</th><th>Length (change in z-score) n=321</th><th>HC (change in z-score) n=321</th></tr>
<tr><td></td><td>Parameter estimate (SE)</td><td>Parameter estimate (SE)</td><td>Parameter estimate (SE)</td></tr>
<tr><td>Growth for reference infant[1]</td><td>-0.216 (0.029) P <0.001[2]</td><td>-0.333 (0.027) P <0.001[2]</td><td>0.013 (0.030) P = 0.654[2]</td></tr>
<tr><td>Proportion formula[3]</td><td>0.018 (0.007) P = 0.009[2]</td><td>0.016 (0.006) P = 0.01[2]</td><td>0.013 (0.007) P = 0.057[2]</td></tr>
</table>
[1] Average monthly change in z-score for reference subjects who received 100% MOM (n=255) at mean NICU LOS, GA and sex.
[2] P value testing hypothesis that the change in z-score is zero
[3] Deviation from the reference growth trend for each week (NICU LOS GA) female sex and for each 10% increase in proportion of PTF
</td>
</tr>
</tbody>
</table>

<table>
<tr><th>Study</th><th>Variable synthesised narratively</th><th>Reason for non-inclusion in meta-analyses</th><th>Comparison for this review</th><th>Results</th></tr>
<tr><td>Jacobi-Polishook, 2016 [5]</td><td>Body mass index gain</td><td>Only study reporting body mass index</td><td>C3</td><td>Body mass index gain
<table>
<tr><th></th><th>Full cohort n=611</th><th>No HM n=46</th><th>Quartile 1 HM n=141</th><th>Quartile 2 HM n=141</th><th>Quartile 3 HM n=142</th><th>Quartile 4 HM n=141</th></tr>
<tr><td>BMI gain, birth to discharge (kg/m^2/wk)</td><td>0.47 ± 0.20</td><td>0.52 ± 0.16</td><td>0.49 ± 0.21</td><td>0.44 ± 0.24</td><td>0.44 ± 0.16</td><td>0.50 ± 0.20</td></tr>
</table></td></tr>
<tr><td>Kaempf 1998 [6]</td><td>Length gain</td><td>Measured lower leg length</td><td>C3</td><td>Lower leg length growth
<table>
<tr><th></th><th>Fortified HM n=8</th><th>PTF n=11</th></tr>
<tr><td>Lower leg length growth velocity (mean ± SD mm/d)</td><td>0.51 ± 0.04</td><td>0.54 ± 0.09</td></tr>
</table></td></tr>
<tr><td>Li 2019 [7]</td><td>Change in wt, length and HC z-scores
Fat mass (g)
Fat-free mass (g)</td><td>Multivariable regression used for association between feed exposure and outcomes.</td><td>C3</td><td>Regression analysis of HM intake and growth outcomes
<table>
<tr><th></th><th>EHM n=56</th><th>High dose HM (predominantly HM) n=39</th><th>Low dose HM (predominantly formula) n= 38</th></tr>
<tr><td>Wt z-score change</td><td>Reference</td><td>0.2 (−0.2 to 0.6), P=0.32</td><td>0.6 (0.2 to 0.1), P<0.01</td></tr>
<tr><td>Length z-score change</td><td>Reference</td><td>0.5 (−0.1 to 1.2), P=0.13</td><td>0.3 (−0.5 to 1.0), P=0.48</td></tr>
<tr><td>HC z-score change</td><td>Reference</td><td>−0.1 (−0.9 to 0.6), P=0.73</td><td>0 (−0.9 to 0.8), P=0.94</td></tr>
<tr><td>FM, g</td><td>Reference</td><td>11.2 (−61.9 to 84.4), P=0.76</td><td>26.1 (−49.2 to 101.5), P=0.50</td></tr>
<tr><td>FFM, g</td><td>Reference</td><td>86.1 (−28.8 to 201.1), P=0.14</td><td>257.4 (139.1 to 375.7), P<0.01</td></tr>
</table>
Data are adjusted mean difference with 95% CIs compared to reference infant fed EHM (n=56) adjusted for covariates including baseline characteristics, age at imaging, total nutrient intake before 34 weeks PMA, and level of care</td></tr>
<tr><td>Manea 2016 [8]</td><td>Wt gain</td><td>SD not reported</td><td>C1</td><td>Weight gain of infants with BW <1kg, within the first 5 weeks of life, according to type of early enteral nutrition given (g/d)
<table>
<tr><th></th><th>Average wt gain</th></tr>
<tr><td>EHM (n= 18)</td><td>17.26</td></tr>
<tr><td>EPTF (n=16)</td><td>13.89</td></tr>
</table>
No SD or p values reported</td></tr>
<tr><td>Nicholl 1999 [9]</td><td>Length gain</td><td>Measured lower leg length</td><td>C1</td><td>Lower leg length velocity – between group change, mean ± SD
<table>
<tr><th></th><th>EHM (Gp2)</th><th>EPTF (Gp3)</th><th></th></tr>
<tr><td>Lower leg length gain (mm/d)</td><td>0.17 ± 0.38</td><td>0.28 ± 0.50</td><td>P=0.9</td></tr>
</table></td></tr>
</table>

Study	Variable synthesised narratively	Reason for non-inclusion in meta-analyses	Comparison for this review	Results
Soldateli 2020 [10]	Wt gain (g/kg/day), change in weight and length z-scores	SD not reported	C3	Human milk feeding and growth to discharge or transfer

	Days fed any HM					P values	
	0-25% n=40	26-50% n=151	51-75% n=268	76-99% n=375	All HM n=594	Across all Groups	Between 0-25% and All HM
Wt gain, g/kg/d	13.8	13.5	13.5	13.7	13.6	0.6	0.3
Change in wt z-score	-0.6	-0.7	-0.7	-0.6	-0.7	0.7	0.2
HC gain, cm/wk	0.8	0.8	0.8	0.8	0.8	0.4	0.1
Change in HC z-score	-0.1	-0.3	-0.4	-0.3	-0.3	0.8	0.2
Change in length z-score	-0.9	-1.3	-1.3	-1.2	-1.1	0.09	0.2

Diet recorded on days of life 7, 14, 21, 28, 42, 56, 70, 84, and at NICU discharge or transfer

Abbreviations: DHM, donor human milk; EHM, exclusive human milk; EPTF, exclusive preterm formula; GA, gestational age; HC, head circumference; HM, human milk; LOS, length of stay; MD, mean difference; MOM, mother's own milk; NICU, neonatal intensive care unit; PTF, preterm formula; SD, standard deviation; SE, standard error; Wt, weight

Table S2. Summary of findings: Preterm formula vs Human milk – Weight gain (g/d)

**Outcomes**	**Anticipated absolute effects* (95% CI)**		**Relative effect (95% CI)**	**№ of participants (studies)**	**Certainty of the evidence (GRADE)**
	**Risk with human milk (exclusive, any or high dose)**	**Risk with preterm formula**			
**Intervention:** Exclusive preterm formula<br>**Comparison:** Exclusive human milk<br>**Study design:** RCT	-	MD 2 higher (1.54 lower to 5.54 higher)	-	53 (1 RCT)	Not graded as only one study. ROB moderate
**Intervention:** Lower dose human milk<br>**Comparison:** Higher dose human milk<br>**Study design: Observational**	-	MD 0.83 lower (1.65 lower to 0)	-	1606 (2 observational studies)	⊕⊕⊖⊖<br>LOW[1]

*The risk in the intervention group (and its 95% confidence interval) is based on the assumed risk in the comparison group and the relative effect of the intervention (and its 95% CI).
Abbreviations: CI, Confidence interval; MD, mean difference; RCT, randomised controlled trial; ROB, risk of bias

[1]Observational studies considered low quality evidence

GRADE Working Group grades of evidence
High certainty: We are very confident that the true effect lies close to that of the estimate of the effect
Moderate certainty: We are moderately confident in the effect estimate: The true effect is likely to be close to the estimate of the effect, but there is a possibility that it is substantially different
Low certainty: Our confidence in the effect estimate is limited: The true effect may be substantially different from the estimate of the effect
Very low certainty: We have very little confidence in the effect estimate: The true effect is likely to be substantially different from the estimate of effect

Table S3. Summary of findings: Preterm formula vs Human milk – Weight gain (g/kg/d)

Outcomes	Anticipated absolute effects* (95% CI)		Relative effect (95% CI)	№ of participants (studies)	Certainty of the evidence (GRADE)
	Risk with human milk (exclusive, any or high dose)	Risk with preterm formula			
**Intervention:** Exclusive preterm formula<br>**Comparison:** Exclusive human milk<br>**Study design:** Observational	-	MD 2.03 higher<br>(0.31 lower to 4.38 higher)	-	364<br>(4 observational studies)	⊕⊖⊖⊖<br>VERY LOW [1 2 3]
**Intervention:** Exclusive preterm formula<br>**Comparison:** Any human milk<br>**Study design:** Observational	-	MD 1.97 higher<br>(0.21 higher to 3.72 higher)	-	795<br>(5 observational studies)	⊕⊖⊖⊖<br>VERY LOW [1 2 3]
**Intervention:** Lower dose human milk<br>**Comparison:** Higher dose human milk<br>**Study design:** RCT	-	MD 2.41 higher<br>(1.09 higher to 3.72 higher)	-	373<br>(2 RCTs)	⊕⊕⊖⊖<br>LOW [3 4]
**Intervention:** Lower dose human milk<br>**Comparison:** Higher dose human milk<br>**Study design:** Observational	-	MD 0.56 higher<br>(0.09 higher to 1.03 higher)	-	3162<br>(13 observational studies)	⊕⊖⊖⊖<br>VERY LOW [1 5]

*The risk in the intervention group (and its 95% confidence interval) is based on the assumed risk in the comparison group and the relative effect of the intervention (and its 95% CI).
Abbreviations: CI, Confidence interval; MD, mean difference; RCT, randomised controlled trial; ROB, risk of bias

[1]Observational studies considered low quality evidence
[2]Downgraded for inconsistency – considerable heterogeneity (I^2 75-100%)
[3]Downgraded for imprecision
[4]Downgraded for ROB – one RCT with high ROB, one RCT with low ROB
[5]Downgraded for inconsistency – substantial heterogeneity (I^2 50-74%)

GRADE Working Group grades of evidence
High certainty: We are very confident that the true effect lies close to that of the estimate of the effect
Moderate certainty: We are moderately confident in the effect estimate: The true effect is likely to be close to the estimate of the effect, but there is a possibility that it is substantially different
Low certainty: Our confidence in the effect estimate is limited: The true effect may be substantially different from the estimate of the effect
Very low certainty: We have very little confidence in the effect estimate: The true effect is likely to be substantially different from the estimate of effect

Table S4. Summary of findings: Preterm formula vs Human milk – change in weight z-score

Outcomes	Anticipated absolute effects* (95% CI)		Relative effect (95% CI)	№ of participants (studies)	Certainty of the evidence (GRADE)
	Risk with human milk (exclusive, any or high dose)	Risk with preterm formula			
**Intervention:** Exclusive preterm formula<br>**Comparison:** Exclusive human milk<br>**Study design:** Observational	-	MD 0.26 higher<br>(0.03 higher to 0.48 higher)	-	494<br>(2 observational studies)	⊕⊕⊖⊖<br>LOW [1]
**Intervention:** Exclusive preterm formula<br>**Comparison:** Any human milk<br>**Study design:** Observational	-	MD 0.21 higher<br>(0.15 lower to 0.56 higher)	-	1532<br>(3 observational studies)	⊕⊖⊖⊖<br>VERY LOW [1 2]
**Intervention:** Lower dose human milk<br>**Comparison:** Higher dose human milk<br>**Study design:** RCT	-	MD 0<br>(0.29 lower to 0.29 higher)	-	326<br>(1 RCT)	Not graded as only one study. ROB low
**Intervention:** Lower dose human milk<br>**Comparison:** Higher dose human milk<br>**Study design:** Observational	-	MD 0.19 higher<br>(0.06 higher to 0.33 higher)	-	4059<br>(12 observational studies)	⊕⊖⊖⊖<br>VERY LOW [1 2]

*The risk in the intervention group (and its 95% confidence interval) is based on the assumed risk in the comparison group and the relative effect of the intervention (and its 95% CI).
Abbreviations: CI, Confidence interval; MD, mean difference; RCT, randomised controlled trial; ROB, risk of bias

[1]Observational studies considered low quality evidence
[2]Downgraded for inconsistency – considerable heterogeneity (I^2 75-100%)

GRADE Working Group grades of evidence
High certainty: We are very confident that the true effect lies close to that of the estimate of the effect
Moderate certainty: We are moderately confident in the effect estimate: The true effect is likely to be close to the estimate of the effect, but there is a possibility that it is substantially different
Low certainty: Our confidence in the effect estimate is limited: The true effect may be substantially different from the estimate of the effect
Very low certainty: We have very little confidence in the effect estimate: The true effect is likely to be substantially different from the estimate of effect

Table S5. Summary of findings: Preterm formula vs Human milk - head circumference gain (cm/wk)

Outcomes	Anticipated absolute effects* (95% CI)		Relative effect (95% CI)	№ of participants (studies)	Certainty of the evidence (GRADE)
	Risk with human milk (exclusive, any or high dose)	Risk with preterm formula			
**Intervention:** Exclusive preterm formula<br>**Comparison:** Exclusive human milk<br>**Study design:** RCT	-	MD 0.1 higher<br>(0.02 lower to 0.22 higher)	-	53<br>(1 RCT)	Not graded as only one study. ROB moderate
**Intervention:** Exclusive preterm formula<br>**Comparison:** Exclusive human milk<br>**Study design:** Observational	-	MD 0.09 higher<br>(0.1 lower to 0.29 higher)	-	78<br>(2 observational studies)	⨁⊝⊝⊝<br>VERY LOW [1 2 3]
**Intervention:** Exclusive preterm formula<br>**Comparison:** Any human milk<br>**Study design:** Observational	-	MD 0.06 higher<br>(0.01 higher to 0.11 higher)	-	495<br>(4 observational studies)	⨁⨁⊝⊝<br>LOW [1]
**Intervention:** Lower dose human milk<br>**Comparison:** Higher dose human milk<br>**Study design:** RCT	-	MD 0<br>(0.06 lower to 0.06 higher)	-	373<br>(2 RCTs)	⨁⨁⨁⊝<br>MODERATE [4]
**Intervention:** Lower dose human milk<br>**Comparison:** Higher dose human milk<br>**Study design:** Observational	-	MD 0.04 higher<br>(0.02 higher to 0.07 higher)	-	4080<br>(10 observational studies)	⨁⊝⊝⊝<br>VERY LOW [5]

*The risk in the intervention group (and its 95% confidence interval) is based on the assumed risk in the comparison group and the relative effect of the intervention (and its 95% CI).
Abbreviations: CI, Confidence interval; MD, mean difference; RCT, randomised controlled trial; ROB, risk of bias

[1]Observational studies considered low quality evidence
[2]Downgraded for inconsistency – considerable heterogeneity (I^2 75-100%)
[3]Downgraded for imprecision
[4]Downgraded for ROB – one RCT with high ROB, one RCT with low ROB
[5]Downgraded for inconsistency – substantial heterogeneity (I^2 50-74%)

GRADE Working Group grades of evidence
High certainty: We are very confident that the true effect lies close to that of the estimate of the effect
Moderate certainty: We are moderately confident in the effect estimate: The true effect is likely to be close to the estimate of the effect, but there is a possibility that it is substantially different
Low certainty: Our confidence in the effect estimate is limited: The true effect may be substantially different from the estimate of the effect
Very low certainty: We have very little confidence in the effect estimate: The true effect is likely to be substantially different from the estimate of effect

Table S6. Summary of findings: Preterm formula vs Human milk – change in head circumference z-score

Outcomes	Anticipated absolute effects* (95% CI)		Relative effect (95% CI)	№ of participants (studies)	Certainty of the evidence (GRADE)
	Risk with human milk (exclusive, any or high dose)	Risk with preterm formula			
**Intervention:** Exclusive preterm formula<br>**Comparison:** Exclusive human milk<br>**Study design:** Observational	-	MD 0.1 higher (0.42 lower to 0.62 higher)	-	32 (1 observational study)	Not graded as only one study. ROB low
**Intervention:** Exclusive preterm formula<br>**Comparison:** Any human milk<br>**Study design:** Observational	-	MD 0.43 higher (0.18 higher to 0.69 higher)	-	322 (2 observational studies)	⊕⊕⊖⊖ LOW [1]
**Intervention:** Lower dose human milk<br>**Comparison:** Higher dose human milk<br>**Study design:** RCT	-	MD 0.2 higher (0.08 lower to 0.48 higher)	-	326 (1 RCT)	Not graded as only one study. ROB low
**Intervention:** Lower dose human milk<br>**Comparison:** Higher dose human milk<br>**Study design:** Observational	-	MD 0.09 higher (0.19 lower to 0.38 higher)	-	2627 (8 observational studies)	⊕⊖⊖⊖ VERY LOW [1 2 3]

*The risk in the intervention group (and its 95% confidence interval) is based on the assumed risk in the comparison group and the relative effect of the intervention (and its 95% CI).
Abbreviations: CI, Confidence interval; MD, mean difference; RCT, randomised controlled trial; ROB, risk of bias

[1]Observational studies considered low quality evidence
[2]Downgraded for inconsistency – considerable heterogeneity (I^2 75-100%)
[3]Downgraded for imprecision

GRADE Working Group grades of evidence
High certainty: We are very confident that the true effect lies close to that of the estimate of the effect
Moderate certainty: We are moderately confident in the effect estimate: The true effect is likely to be close to the estimate of the effect, but there is a possibility that it is substantially different
Low certainty: Our confidence in the effect estimate is limited: The true effect may be substantially different from the estimate of the effect
Very low certainty: We have very little confidence in the effect estimate: The true effect is likely to be substantially different from the estimate of effect

Table S7. Summary of findings: Preterm formula vs Human milk – length gain (cm/week)

Outcomes	Anticipated absolute effects* (95% CI)		Relative effect (95% CI)	№ of participants (studies)	Certainty of the evidence (GRADE)
	Risk with human milk (exclusive, any or high dose)	Risk with preterm formula			
**Intervention:** Exclusive preterm formula<br>**Comparison:** Exclusive human milk<br>**Study design:** RCT	-	MD 0.28 higher (0.14 higher to 0.42 higher)	-	53 (1 RCT)	Not graded as only one study. ROB moderate
**Intervention:** Exclusive preterm formula<br>**Comparison:** Exclusive human milk<br>**Study design:** Observational	-	MD 0.06 higher (0.07 lower to 0.19 higher)	-	78 (2 observational studies)	⨁⊖⊖⊖<br>VERY LOW [1 2]
**Intervention:** Exclusive preterm formula<br>**Comparison:** Any human milk<br>**Study design:** Observational	-	MD 0.09 higher (0.05 lower to 0.22 higher)	-	778 (3 observational studies)	⨁⊖⊖⊖<br>VERY LOW [1 2 3]
**Intervention:** Lower dose human milk<br>**Comparison:** Higher dose human milk<br>**Study design:** RCT	-	MD -0.04 (0.28 lower to 0.21 higher)	-	373 (2 RCTs)	⨁⨁⊖⊖<br>LOW [1 4 5]
**Intervention:** Lower dose human milk<br>**Comparison:** Higher dose human milk<br>**Study design:** Observational	-	MD 0.05 higher (0.02 higher to 0.08 higher)	-	2423 (8 observational studies)	⨁⨁⊖⊖<br>LOW [1]

*The risk in the intervention group (and its 95% confidence interval) is based on the assumed risk in the comparison group and the relative effect of the intervention (and its 95% CI).
Abbreviations: CI, Confidence interval; MD, mean difference; RCT, randomised controlled trial; ROB, risk of bias

[1]Observational studies considered low quality evidence
[2]Downgraded for imprecision
[3]Downgraded for inconsistency – considerable heterogeneity (I^2 75-100%)
[4]Downgraded for ROB – one RCT with high ROB, one RCT with low ROB
[5]Downgraded for inconsistency – substantial heterogeneity (I^2 50-74%)

GRADE Working Group grades of evidence
High certainty: We are very confident that the true effect lies close to that of the estimate of the effect
Moderate certainty: We are moderately confident in the effect estimate: The true effect is likely to be close to the estimate of the effect, but there is a possibility that it is substantially different
Low certainty: Our confidence in the effect estimate is limited: The true effect may be substantially different from the estimate of the effect
Very low certainty: We have very little confidence in the effect estimate: The true effect is likely to be substantially different from the estimate of effect

Table S8. Summary of findings: Preterm formula vs Human milk - change in length z-score

Outcomes	Anticipated absolute effects* (95% CI)		Relative effect (95% CI)	№ of participants (studies)	Certainty of the evidence (GRADE)
	Risk with human milk (exclusive, any or high dose)	Risk with preterm formula			
**Intervention:** Exclusive preterm formula<br>**Comparison:** Exclusive human milk<br>**Study design:** Observational	-	MD 0<br>(0.63 lower to 0.63 higher)	-	32<br>(1 observational study)	Not graded as only one study. ROB low
**Intervention:** Lower dose human milk<br>**Comparison:** Higher dose human milk<br>**Study design:** RCT	-	MD 0.1 higher<br>(0.26 lower to 0.46 higher)	-	326<br>(1 RCT)	Not graded as only one study. ROB low
**Intervention:** Lower dose human milk<br>**Comparison:** Higher dose human milk<br>**Study design:** Observational	-	MD 0.09 higher<br>(0.07 lower to 0.25 higher)	-	1131<br>(3 observational studies)	⨁⊖⊖⊖<br>VERY LOW [1 2 3]

*The risk in the intervention group (and its 95% confidence interval) is based on the assumed risk in the comparison group and the relative effect of the intervention (and its 95% CI).
Abbreviations: CI, Confidence interval; MD, mean difference; RCT, randomised controlled trial; ROB, risk of bias

[1]Observational studies considered low quality evidence
[2]Downgraded for inconsistency – considerable heterogeneity (I^2 75-100%)
[3]Downgraded for imprecision

GRADE Working Group grades of evidence
High certainty: We are very confident that the true effect lies close to that of the estimate of the effect
Moderate certainty: We are moderately confident in the effect estimate: The true effect is likely to be close to the estimate of the effect, but there is a possibility that it is substantially different
Low certainty: Our confidence in the effect estimate is limited: The true effect may be substantially different from the estimate of the effect
Very low certainty: We have very little confidence in the effect estimate: The true effect is likely to be substantially different from the estimate of effect

Table S9. Summary of findings: Preterm formula vs Human milk - % fat-free mass

Outcomes	Anticipated absolute effects* (95% CI)		Relative effect (95% CI)	№ of participants (studies)	Certainty of the evidence (GRADE)
	Risk with human milk (exclusive, any or high dose)	Risk with preterm formula			
**Intervention:** Exclusive preterm formula<br>**Comparison:** Exclusive human milk<br>**Study design:** Observational	-	MD 1.46 lower<br>(4.35 lower to 1.43 higher)	-	87<br>(3 observational studies)	⨁⊖⊖⊖<br>VERY LOW [1 2]
**Intervention:** Lower dose human milk<br>**Comparison:** Higher dose human milk<br>**Study design:** Observational	-	MD 5.1 lower<br>(12.45 lower to 2.25 higher)	-	73<br>(1 observational study)	Not graded as only one study. ROB low

*The risk in the intervention group (and its 95% confidence interval) is based on the assumed risk in the comparison group and the relative effect of the intervention (and its 95% CI).
Abbreviations: CI, Confidence interval; MD, mean difference; RCT, randomised controlled trial; ROB, risk of bias

[1]Observational studies considered low quality evidence
[2]Downgraded for inconsistency – considerable heterogeneity (I^2 75-100%)

GRADE Working Group grades of evidence
High certainty: We are very confident that the true effect lies close to that of the estimate of the effect
Moderate certainty: We are moderately confident in the effect estimate: The true effect is likely to be close to the estimate of the effect, but there is a possibility that it is substantially different
Low certainty: Our confidence in the effect estimate is limited: The true effect may be substantially different from the estimate of the effect
Very low certainty: We have very little confidence in the effect estimate: The true effect is likely to be substantially different from the estimate of effect

Table S10. Summary of findings: Preterm formula vs Human milk - fat-free mass (g)

Outcomes	Anticipated absolute effects* (95% CI)		Relative effect (95% CI)	№ of participants (studies)	Certainty of the evidence (GRADE)
	Risk with human milk (exclusive, any or high dose)	Risk with preterm formula			
**Intervention:** Exclusive preterm formula<br>**Comparison:** Exclusive human milk<br>**Study design:** Observational	-	MD 130.18 higher (53.86 higher to 206.5 higher)	-	134 (4 observational studies)	⨁⊖⊖⊖ VERY LOW [1 2]

*The risk in the intervention group (and its 95% confidence interval) is based on the assumed risk in the comparison group and the relative effect of the intervention (and its 95% CI).
Abbreviations: CI, Confidence interval; MD, mean difference

[1]Observational studies considered low quality evidence
[2]Downgraded for imprecision

GRADE Working Group grades of evidence
High certainty: We are very confident that the true effect lies close to that of the estimate of the effect
Moderate certainty: We are moderately confident in the effect estimate: The true effect is likely to be close to the estimate of the effect, but there is a possibility that it is substantially different
Low certainty: Our confidence in the effect estimate is limited: The true effect may be substantially different from the estimate of the effect
Very low certainty: We have very little confidence in the effect estimate: The true effect is likely to be substantially different from the estimate of effect

Table S11. Summary of findings: Preterm formula vs Human milk - % fat mass

Outcomes	Anticipated absolute effects* (95% CI)		Relative effect (95% CI)	№ of participants (studies)	Certainty of the evidence (GRADE)
	Risk with human milk (exclusive, any or high dose)	Risk with preterm formula			
**Intervention:** Exclusive preterm formula<br>**Comparison:** Exclusive human milk<br>**Study design:** Observational	-	MD 1.82 higher (0.59 lower to 4.23 higher)	-	141 (4 observational studies)	⨁⊖⊖⊖ VERY LOW [1 2]
**Intervention:** Lower dose human milk<br>**Comparison:** Higher dose human milk<br>**Study design:** Observational	-	MD 0.48 lower (1.7 lower to 0.73 higher)	-	133 (1 observational study)	Not graded as only one study. ROB low

*The risk in the intervention group (and its 95% confidence interval) is based on the assumed risk in the comparison group and the relative effect of the intervention (and its 95% CI).
Abbreviations: CI, Confidence interval; MD, mean difference; ROB, risk of bias

[1]Observational studies considered low quality evidence
[2]Downgraded for inconsistency – considerable heterogeneity (I^2 75-100%)

GRADE Working Group grades of evidence
High certainty: We are very confident that the true effect lies close to that of the estimate of the effect
Moderate certainty: We are moderately confident in the effect estimate: The true effect is likely to be close to the estimate of the effect, but there is a possibility that it is substantially different
Low certainty: Our confidence in the effect estimate is limited: The true effect may be substantially different from the estimate of the effect
Very low certainty: We have very little confidence in the effect estimate: The true effect is likely to be substantially different from the estimate of effect

Table S12. Summary of findings: Preterm formula vs Human milk - fat mass (g)

Outcomes	Anticipated absolute effects* (95% CI)		Relative effect (95% CI)	№ of participants (studies)	Certainty of the evidence (GRADE)
	Risk with human milk (exclusive, any or high dose)	Risk with preterm formula			
**Intervention:** Exclusive preterm formula<br>**Comparison:** Exclusive human milk<br>**Study design:** Observational	-	MD 60.94 higher (5.42 lower to 127.31 higher)	-	134 (4 observational studies)	⨁⊖⊖⊖ VERY LOW [1 2 3]

*The risk in the intervention group (and its 95% confidence interval) is based on the assumed risk in the comparison group and the relative effect of the intervention (and its 95% CI).
Abbreviations: CI, Confidence interval; MD, mean difference

[1]Observational studies considered low quality evidence
[2]Downgraded for inconsistency – considerable heterogeneity (I^2 75-100%)
[3]Downgraded for imprecision

GRADE Working Group grades of evidence
High certainty: We are very confident that the true effect lies close to that of the estimate of the effect
Moderate certainty: We are moderately confident in the effect estimate: The true effect is likely to be close to the estimate of the effect, but there is a possibility that it is substantially different
Low certainty: Our confidence in the effect estimate is limited: The true effect may be substantially different from the estimate of the effect
Very low certainty: We have very little confidence in the effect estimate: The true effect is likely to be substantially different from the estimate of effect

Figures

Figure S1. Prisma diagram – selection of studies

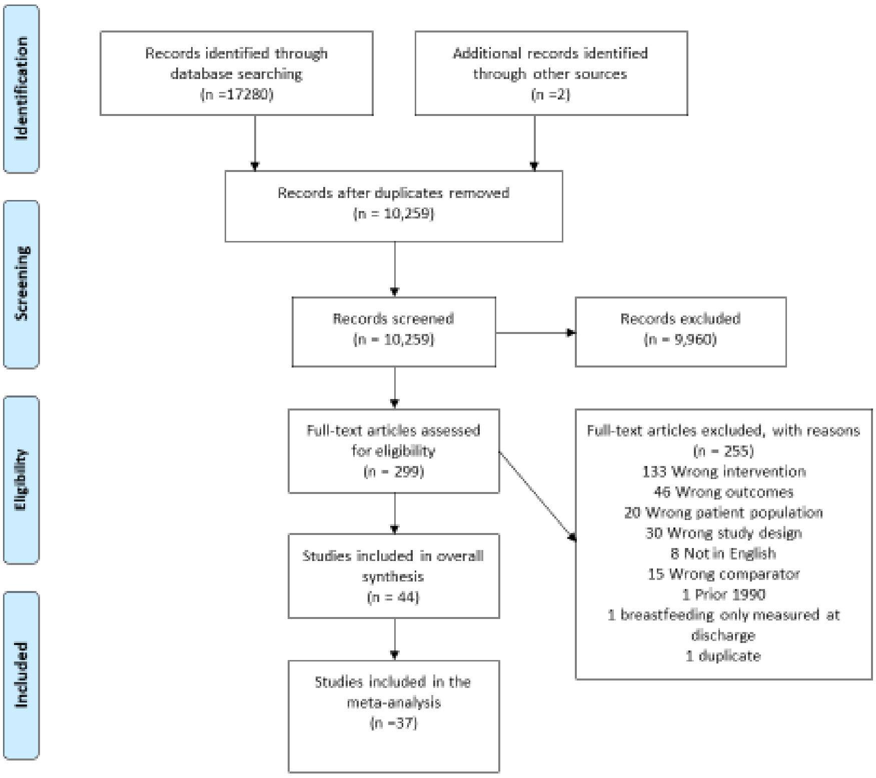

References

1. Brownell, E.A.; Matson, A.P.; Smith, K.C.; Moore, J.E.; Esposito, P.A.; Lussier, M.M.; Lerer, T.J.; Hagadorn, J.I. Dose-response relationship between donor human milk, mother's own milk, preterm formula, and neonatal growth outcomes. *J. Pediatr. Gastroenterol. Nutr.* **2018**, *67*, 90-96.
2. Castellano Yáñez, C.; Castillo Barrio, B.; Muñoz Labián, M.D.C.; Ortiz Movilla, R.; García Lara, N.R.; Royuela Vicente, A.; Marín Gabriel, M.A. Providing very preterm infants with donor human milk led to faster breastfeeding rates but worse biometric gains. *Acta. Paediatr.* **2019**, *108*, 766-767.
3. Carlson, S.J.; Ziegler, E.E. Nutrient intakes and growth of very low birth weight infants. *J. Perinatol.* **1998**, *18*, 252-258.
4. Hoban, R.; Schoeny, M.E.; Esquerra-Zwiers, A.; Kaenkumchorn, T.K.; Casini, G.; Tobin, G.; Siegel, A.H.; Patra, K.; Hamilton, M.; Wicks, J., *et al.* Impact of donor milk on short- and long-term growth of very low birth weight infants. *Nutrients* **2019**, *11*.
5. Jacobi-Polishook, T.; Collins, C.T.; Sullivan, T.R.; Simmer, K.; Gillman, M.W.; Gibson, R.A.; Makrides, M.; Belfort, M.B. Human milk intake in preterm infants and neurodevelopment at 18 months corrected age. *Pediatr. Res.* **2016**.
6. Kaempf, D.E.; Pfluger, M.S.; Thiele, A.M.; Hermanussen, M.; Linderkamp, O. Influence of nutrition on growth in premature infants: Assessment by knemometry. *Ann. Hum. Biol.* **1998**, *25*, 127-136.
7. Li, Y.; Liu, X.; Modi, N.; Uthaya, S. Impact of breast milk intake on body composition at term in very preterm babies: Secondary analysis of the nutritional evaluation and optimisation in neonates randomised controlled trial. *Arch. Dis. Child. Fetal. Neonatal Ed.* **2019**, *104*, F306-f312.
8. Manea, A.; Boia, M.; Iacob, D.; Dima, M.; Iacob, R.E. Benefits of early enteral nutrition in extremely low birth weight infants. *Singapore Med. J.* **2016**, *57*, 616-618.
9. Nicholl, R.M.; Gamsu, H.R. Changes in growth and metabolism in very low birthweight infants fed with fortified breast milk. *Acta. Paediatr.* **1999**, *88*, 1056-1061.
10. Soldateli, B.; Parker, M.; Melvin, P.; Gupta, M.; Belfort, M. Human milk feeding and physical growth in very low-birth-weight infants: A multicenter study. *J. Perinatol.* **2020**, *40*, 1246-1252.
